# ZDHHC12 Palmitoylates HDAC8 to Promote the Progression of Hepatocellular Carcinoma Associated with a Diet High in Saturated Fatty Acids

**DOI:** 10.1002/advs.202505702

**Published:** 2025-08-11

**Authors:** Xin Jin, Yulong Hong, Yongqi Zhao, Wensheng Shi, Ruilin Liu, Xinyu You, Chong Yang, Yu Zhang

**Affiliations:** ^1^ Key Laboratory of Diabetes Immunology (Central South University) Ministry of Education National Clinical Research Center for Metabolic Disease Changsha Hunan 410011 China; ^2^ Uro‐Oncology Institute of Central South University Changsha Hunan 410011 China; ^3^ Hunan Key Laboratory of Tumor Models and Individualized Medicine The Second Xiangya Hospital Central South University Changsha Hunan 410011 China; ^4^ Hepatobiliary and Pancreatic Surgery Department Sichuan Provincial People's Hospital University of Electronic Science and Technology of China Chengdu Sichuan 611731 China

**Keywords:** HCC, HDAC8, palmitic acid, ZDHHC12

## Abstract

Excessive intake of saturated fatty acids (SFAs)—commonly associated with diets rich in fried foods and red meat—significantly increases the risk of hepatocellular carcinoma (HCC). Palmitic acid (PA), the most prevalent type of SFA, is selected as the representative model for this study. Palmitoyltransferases play crucial roles in protein palmitoylation mediated by PA; however, the most significantly altered palmitoyltransferase under high‐SFA dietary conditions remains unidentified. This study reveals zinc finger DHHC‐type palmitoyltransferase 12 (ZDHHC12) as a key protein in PA‐driven HCC progression that functions by stabilizing the oncogenic histone deacetylase 8 (HDAC8). Mechanistically, PA supplementation upregulates ZDHHC12 expression by activating the transcription factor SWI/SNF‐related BAF chromatin remodeling complex subunit ATPase 4 (SMARCA4). ZDHHC12 mediates HDAC8 palmitoylation at cysteine 244, thereby inhibiting its lysosomal degradation and ultimately promoting HCC progression. This study reveals that ZDHHC12 is a critical mediator of PA‐induced HCC progression and that targeting HDAC8 can suppress this process. These findings offer a potential therapeutic strategy for HCC patients with high dietary intake of SFAs, particularly PA.

## Graphical abstract

This study identifies ZDHHC12 as a critical mediator of PA‐induced HCC progression. Mechanistically, PA upregulates ZDHHC12 expression through SMARCA4 activation, leading to HDAC8 palmitoylation at Cys244 which blocks HSC70‐mediated lysosomal degradation of HDAC8, thereby promoting HCC development. These findings provide novel mechanistic insights into how high SFA/PA diets accelerate HCC progression.

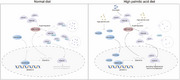

## 1 Introduction

Hepatocellular carcinoma (HCC) remains a major clinical challenge, and its incidence is projected to exceed one million cases by 2025.^[^ ^1^ ^]^ In addition to hepatitis B virus (HBV) infection and alcohol use, nonalcoholic fatty liver disease (NAFLD) is rapidly emerging as a major cause of HCC.^[^ ^2^ ^]^ Saturated fatty acids (SFAs), which are abundant in red meat, pose significant health risks and have been shown to promote the progression of prostate, colorectal, and breast cancers.^[^ ^3^ ^]^ Clinical evidence further indicates that high SFA intake increases the risk of HCC‐related mortality and may serve as a metabolic biomarker for HCC.^[^ ^4^ ^]^ However, current evidence does not conclusively demonstrate a causal association between SFA exposure and HCC. In this study, we employed Mendelian randomization analysis to determine whether there is a causal relationship between SFAs and HCC development.

Palmitic acid (PA) is the most abundant SFA in the diet, accounting for more than half of dietary SFA intake.^[^ ^5^ ^]^ In addition to its involvement in the energy supply, PA can be converted to palmitoyl coenzyme A, thus becoming a substrate for protein S‐palmitoylation.^[^ ^6^ ^]^ Protein function and expression are usually regulated by posttranslational modifications, such as phosphorylation, ubiquitination, glycosylation and palmitoylation.^[^ ^7^ ^]^ Palmitoylation is a reversible posttranslational modification involving the attachment of fatty acids to proteins and may affect 10–20% of the human proteome.^[^ ^8^ ^]^ Owing to differences in tissue specificity or cancer type, palmitoylation can either promote cancer or act as a cancer suppressor, which can be explained by differences in the relative expression of ZDHHC enzymes and key substrates.^[^ ^9^ ^]^ The palmitoylation of many oncogenes promotes cancer progression, such as AKT palmitoylation for hepatocellular carcinoma progression, mTOR palmitoylation for breast cancer progression, and EGFR palmitoylation for lung cancer progression.^[^ ^10^ ^]^ In the tumor microenvironment, the palmitoylation of PD‐L1, PD‐1 and TIM‐3 increases their stability and promotes tumor immune evasion.^[^ ^11^ ^]^ In addition, several studies have shown that protein palmitoylation inhibits the malignant phenotype of cancer cells or enhances cancer immunity.^[^ ^12^ ^]^ Previous studies have shown that the palmitoylation of various proteins (e.g., AKT and PHF2) or increased PA production by O‐GlcNAc transferase can promote HCC progression.^[^ ^10^, ^13^, ^14^ ^]^ However, no studies have identified which specific palmitoyltransferase is significantly upregulated by PA‐enriched red meat consumption and functionally contributes to hepatocarcinogenesis. Moreover, existing mechanistic frameworks fail to adequately explain how this diet promotes HCC progression.

With this study, we demonstrated that ZDHHC12 is markedly induced by PA‐enriched red meat diets and plays a critical role in promoting HCC development. Mechanistically, we identified a novel PA–SMARCA4–ZDHHC12 axis that increases ZDHHC12 expression, and we showed that ZDHHC12 palmitoylates and stabilizes HDAC8, which promotes HCC progression.

## 2 Results

### 2.1 ZDHHC12 Plays a Key Role in PA‐Induced HCC

**Figure 1:**
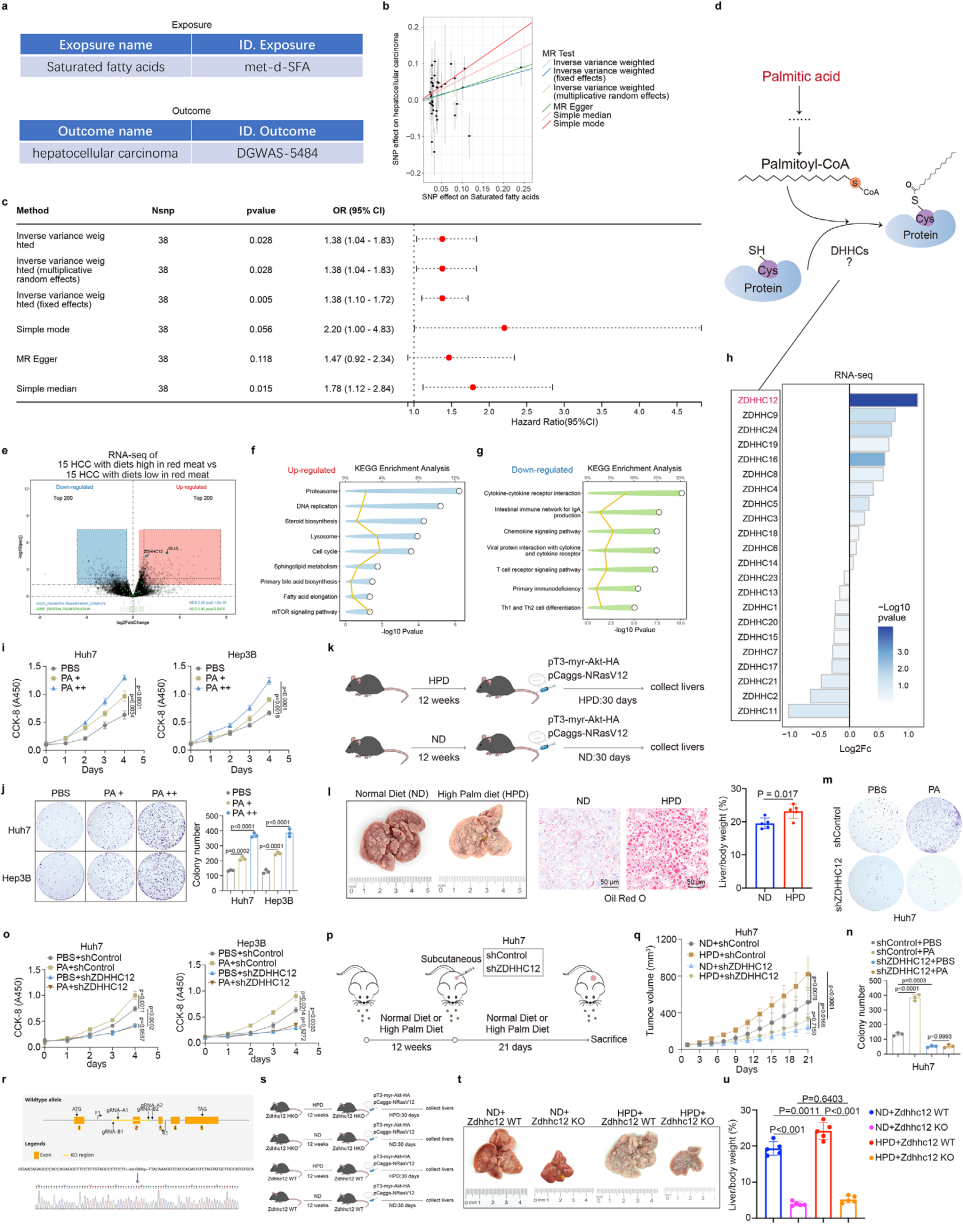
ZDHHC12 plays a key role in PA‐induced HCC. a) both exposure (SFA, ID: met‐d‐SFA) and outcome (HCC, ID: DGWAS‐5484) genetic associations were sourced from summary‐level data in the DMRdb repository (http://www.inbirg.com/DMRdb/). b) Scatter plots of the relationship between SNP effects in SFA and hepatocellular carcinoma obtained by multiple statistical methods. Each point indicates a SNP locus, and the color line indicates the MR fitting result. These results were derived from the DMRdb database (http://www.inbirg.com/DMRdb/). c) Forest plots of causal relationships between exposure variables (SFA) and outcome variables (hepatocellular carcinoma) obtained by multiple statistical methods. d) Pattern diagram for the involvement of palmitic acid in protein palmitoylation modification. e–g) Sequencing results of tissues from 15 HCC patients with high red meat diet and 15 HCC patients with normal diet (e). KEGG enrichment analysis reveals f) up‐regulated and g) down‐regulated pathways in patients with high red meat diet. h) Changes in palmitoyltransferases (DHHCs) in RNA sequencing results from 15 HCC patients with high red meat diet and 15 HCC patients with normal diet. i,j) CCK‐8 and colony formation assays performed on hepatocellular carcinoma cells treated with different concentrations of palmitic acid (+, 100 µm; ++, 200 µm). k,l) Liver cancer was induced by tail vein injection of plasmids in C57BL/6 mice fed with high palmitic acid diet (HPD) or normal diet (ND) for k) 12 weeks. Mouse livers were then collected and photographed, subjected to Oil Red O staining, l) calculation of liver weight ratios. m–o) Cells were transfected with shControl or shZDHHC12 plasmids. Puromycin selection was initiated 72 h post‐transfection. After successful selection, stably transfected cells were collected. m,n) The cells were treated with PBS or palmitic acid (200 µm) and collected for colony formation and o) CCK‐8 assays. p,q) Huh7 cells were transfected with shControl or shZDHHC12. Puromycin selection was initiated 72 h post‐transfection. After successful selection, stably transfected cells were collected and subcutaneously injected into the dorsal flank of nude mice that had been fed a high palmitic acid diet (HPD) or a normal diet (ND) for 12 weeks. Tumor growth curves were plotted, and tumors were harvested 21 d later. r) A schematic diagram of the production of *Zdhhc12* KO mice and a demonstration of the knockout sequence. s–u) Liver cancer was induced by tail vein injection of pT3‐myr‐Akt‐HA and pCaggs‐NRasV12 plasmids in *Zdhhc12* WT and KO mice fed with high palmitic acid diet (HPD) or normal diet (ND) for s) 12 weeks. Mouse livers were then t) collected and photographed, subjected to u) calculation of liver weight ratios.

MR analysis reveals the causal relationship between exposure and the outcome while minimizing the effects of confounding factors and is considered one of the most appropriate methods for studying the causal relationship between exposure and disease.^[^ ^15^ ^]^ We performed Mendelian randomization analyses by using SFAs as an exposure variable and HCC as an outcome variable (**Figure** **1**a). The results revealed that SFA exposure contributes to HCC risk (Figure 1b,c). PA is the most common type of SFA.^[^ ^5^ ^]^ PA generates palmitoyl CoA, which ultimately relies on palmitoyltransferases (DHHCs) that participate in the palmitoylation of proteins to regulate their function^[^ ^6^ ^]^ (Figure 1d). Patients with high red meat intake are commonly used in studies to represent patients on high‐SFA diets.^[^ ^16^ ^]^ Transcriptome sequencing analysis of 15 HCC patients who consumed a diet high in red meat and 15 HCC patients who consumed a normal diet revealed significant enrichment of palmitoylation modification‐related pathways in the former group (Figure 1e); ZDHHC12 was the most significantly upregulated enzyme among all DHHCs (Figure 1h). Subsequent enrichment analysis revealed that fatty acid metabolism‐related pathways and various procancer pathways were upregulated in HCC patients who consumed a diet high in red meat (Figure 1f), whereas antitumor immune‐related functions were suppressed (Figure 1g). We subsequently found that PA, the most common SFA, promoted the proliferation of HCC cells in vitro (Figure 1i,j). Compared with normal diet‐fed HCC model mice, those fed a high‐PA diet presented a significant increase in lipid content and a greater proportion of tumors in the liver (Figure 1k,l). Because PA is the most common SFA and our results revealed that ZDHHC12 was the most significantly upregulated palmitoyltransferase in HCC patients with a high‐red meat diet, we investigated whether ZDHHC12 was involved in PA‐induced HCC progression. We first observed elevated Zdhhc12 expression in HPD‐fed mice compared with that in ND‐fed controls (Figure S1a,b, Supporting Information). ZDHHC12 was found to be upregulated in HCC tissues from patients, and its level increased with pathological grade (Figure S1c–g, Supporting Information). Furthermore, high ZDHHC12 expression predicted a poor prognosis of HCC (Figure S1h—k, Supporting Information). Our integrated analysis of HCC single‐cell RNA sequencing datasets revealed that ZDHHC12 is expressed predominantly in HCC cells, with particularly high expression observed in the most highly malignant tumor cells (Figure S2a–g, Supporting Information). ZDHHC12 knockdown significantly inhibited the proliferation of HCC cells (Figure S3a—c, Supporting Information). ZDHHC12 promoted HCC progression, but its enzymatically inactive mutant, ZDHHC12 C127S,^[^ ^17^ ^]^ lost its ability to promote HCC progression (Figure S3d—g, Supporting Information). In vitro experiments revealed that ZDHHC12 knockdown attenuated the procancer effect of PA (Figure S3h, Supporting Information and Figure 1m–o). A subcutaneous tumorigenesis assay demonstrated that ZDHHC12 knockdown attenuated the tumor‐promoting effects of a high‐palmitate diet (Figure S3i—k, Supporting Information and Figure 1p,q). We generated complete *Zdhhc12* knockout mice for subsequent studies (Figure 1r and Figure S4a,b, Supporting Information). HCC models were established after wild‐type *Zdhhc12* or knockout mice were fed either a high‐PA diet or a normal diet (Figure 1s and Figure S4c,d, Supporting Information). Our results revealed that the knockout of *Zdhhc12* attenuated the HCC‐promoting effect of a high‐PA diet (Figure 1t,u). Thus, PA, the most common SFA, can promote HCC progression, and ZDHHC12 mediates this process.

### 2.2 PA Upregulates ZDHHC12 via SMARCA4

**Figure 2:**
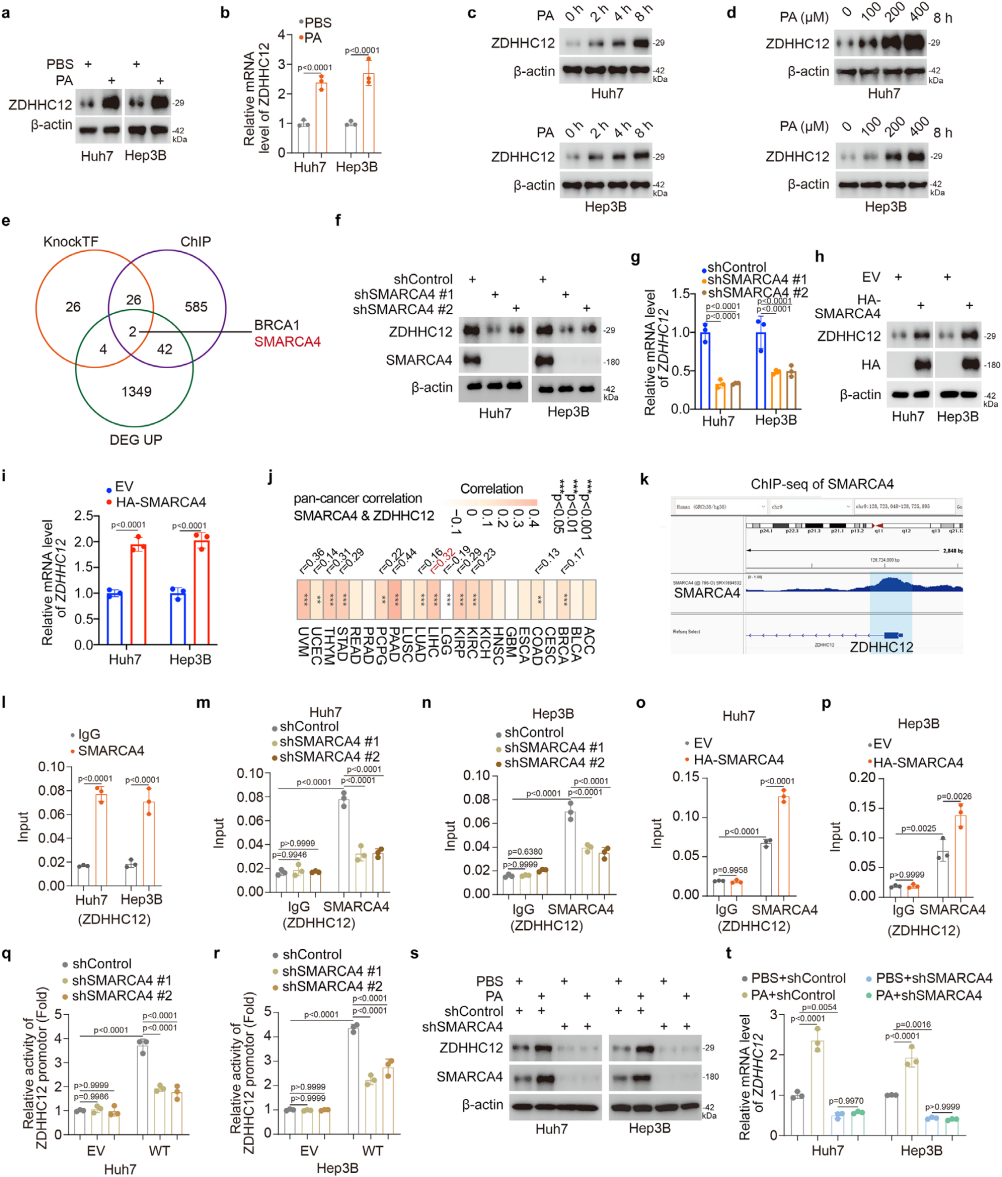
PA upregulates ZDHHC12 via SMARCA4. a,b) Palmitic acid (200 µm, 8 h) was added to hepatocellular carcinoma cells Huh7 and Hep3B for a) western blot and b) RT‐qPCR experiments. c) Hepatocellular carcinoma cells Huh7 and Hep3B were treated with palmitic acid (200 µm) for western blot experiments. d) Western blot experiments were performed by adding different concentrations of palmitic acid (8 h) to hepatocellular carcinoma cells Huh7 and Hep3B. e) The intersection of transcription factors causing changes in ZDHHC12 in KnockTF, proteins binding to the ZDHHC12 promoter in ChIP‐atlas, and up‐regulated genes in 15 cancer tissues was taken to obtain potential transcription factors for ZDHHC12. f,g) Huh7 and Hep3B cells were transfected with the indicated constructs for 72 h and collected for f) western blot and g) RT‐qPCR assay. h,i) Huh7 and Hep3B cells were transfected with the indicated constructs for 24 h and collected for h) western blot and i) RT‐qPCR assay. j) The analysis was performed using TIMER2.0 (http://timer.cistrome.org/) to examine the correlation between ZDHHC12 and SMARCA4 expression levels in TCGA data. k) Analysis of ChIP‐seq data for SMARCA4 indicated that SMARCA4 binds to the promoter of ZDHHC12. l) Huh7 and Hep3B cells were collected and ChIP‐qPCR analysis was performed using IgG or SMARCA4 antibodies. m,n) Huh7 and Hep3B cells were transfected with indicated constructs for 72 h. m) Huh7 and n) Hep3 cells were collected for ChIP‐qPCR analysis by using the IgG or SMARCA4 antibody. o) Huh7 and p) Hep3B cells were transfected with empty vector or the indicated overexpression plasmids for 24 h, cells were collected for ChIP‐qPCR analysis by using the IgG or SMARCA4 antibody. q,r) Indicated constructs were transfected into q) Huh7 and r) Hep3B cells for 72 h and the cells were collected for luciferase reporter assays. s,t) Huh7 and Hep3B cells were transfected with indicated constructs for 72 h and then were treated with palmitic acid (200 µm) for 8 h. Cells were collected for s) western blot and t) RT‐qPCR analysis.

ZDHHC12 is involved in PA‐promoted HCC progression, but the regulatory mechanism by which PA affects ZDHHC12 remains unknown. Upon the addition of PA to HCC cells, ZDHHC12 was upregulated at both the mRNA and protein levels (**Figure** **2**a,b). ZDHHC12 expression changed with increasing duration of PA treatment and increasing PA concentration (Figure 2c,d). We further investigated the underlying causes of these changes in ZDHHC12 expression. We screened for genes upregulated in HCC tissues and intersected them with the transcription factors from the KNOCKTF database that are known to upregulate ZDHHC12, as well as with the transcription factors that bind to the ZDHHC12 promoter, as identified by ChIP‐seq (Figure 2e). We ultimately identified two genes, BRCA1 and SMARCA4 (Figure 2e), that could be transcription factors for ZDHHC12. Previous studies have shown that lipids promote SMARCA4 expression^[^ ^18^ ^]^ and that SMARCA4 has oncogenic potential in HCC.^[^ ^19^ ^]^ We therefore hypothesized that SMARCA4 is involved in PA‐promoted HCC progression. We found that the knockdown or overexpression of BRCA1 did not affect the expression level of ZDHHC12, but the knockdown or overexpression of SMARCA4 changed the expression level of ZDHHC12 (Figure 2f–i and Figure S5a–d, Supporting Information). Analysis of The Cancer Genome Atlas (TCGA) revealed a positive correlation between SMARCA4 and ZDHHC12 expression levels across multiple cancer types, including HCC (Figure 2j and Figure S5e, Supporting Information). ChIP‒seq and ChIP‒qPCR analyses revealed that SMARCA4 binds to the promoter region of ZDHHC12, and a dual‐luciferase reporter assay demonstrated that SMARCA4 activated the ZDHHC12 promoter (Figure 2k–r and Figure S5f,g, Supporting Information). We found that genes upregulated in the high‐red‐meat diet group were enriched in the Wnt signaling pathway, indicating its activation (Figure S5h, Supporting Information). By intersecting the genes upregulated in this group with transcription factors predicted to regulate SMARCA4 from two independent databases, we identified SP5 as the only potential mediator of this effect (Figure S5i, Supporting Information). Since SP5 is a critical downstream transcriptional effector of Wnt signaling and the DNA fragment between ‐285 and ‐279 in the 5′ flanking region of SP5 is a target of the β‐catenin/Tcf4 complex, we hypothesize that the Wnt‐mediated upregulation of SP5 serves as the mechanistic basis for the increased expression of SMARCA4.^[^ ^20^, ^21^ ^]^ The subsequent experimental results validated this hypothesis. Specifically, Wnt treatment upregulated both SP5 and SMARCA4 (Figure S5j, Supporting Information); PA treatment induced SP5 and SMARCA4 expression via Wnt signaling (Figure S5k, Supporting Information); SP5 directly upregulated SMARCA4 (Figure S5l,m, Supporting Information); and PA increased SMARCA4 expression through SP5‐dependent mechanisms (Figure S5n, Supporting Information). Additionally, ChIP‒qPCR (Figure S5o, Supporting Information) and dual‐luciferase reporter assays (Figure S5p, Supporting Information) demonstrated that SP5 binds to the promoter region of SMARCA4 and activates its transcription. Subsequent western blot and RT‒qPCR experiments revealed that the PA‐induced upregulation of ZDHHC12 was accomplished through SMARCA4 (Figure 2s,t). Our results suggest that PA causes the upregulation of ZDHHC12 through SMARCA4.

### 2.3 HDAC8 Is the Downstream Factor through Which ZDHHC12 Promotes HCC

**Figure 3:**
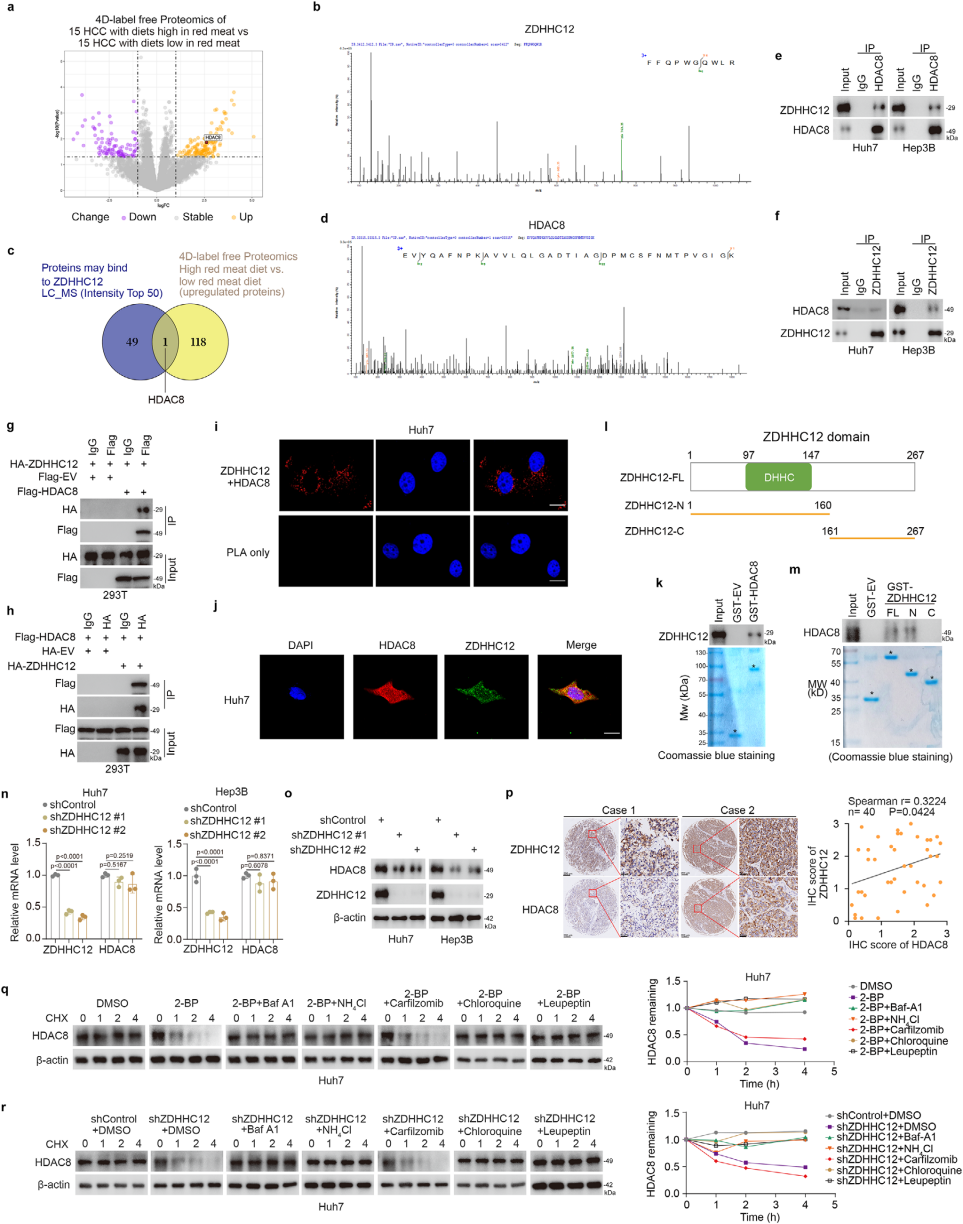
HDAC8 is the downstream factor via which ZDHHC12 promotes HCC. a) HCC tissues from15 HCC patients with high red meat diet and 15 HCC patients with normal diet were collected for 4D‐label free quantitative proteomics assay. b) We performed ZDHHC12 pull‐down and mass spectrometry using Hep3B cells and antibodies to ZDHHC12. c) The intersection results between the top 50 proteins most likely to bind ZDHHC12 (from mass spectrometry data) and proteins upregulated in the high‐red‐meat diet group. d, mass spectrometric peptide mapping of HDAC8. e,f) Huh7 and Hep3B cells were collected for immunoprecipitation assay. g,h) 293T cells were transfected with indicated constructs for 24 h and collected for immunoprecipitation assay. i) The PLA assay was performed in Huh7 cells by using the indicated antibodies. j) Co‐localization analysis of ZDHHC12 and HDAC8 in Huh7 cells. k) GST‐pulled down assay was performed by using the recombinant protein of HDAC8. l) Pattern diagram of constructed ZDHHC12 truncates. m) GST‐pulled down assay was performed by using the recombinant protein of ZDHHC12. n,o) Huh7 and Hep3B cells were transfected with indicated constructs for 72 h and collected for n) RT‐qPCR and o) western blot analysis. p) Representative images and protein IHC score correlation plot of ZDHHC12 and HDAC8 in hepatocellular carcinoma tissue microarrays. q) Degradation of HDAC8 in Huh7 cells treated with or without 2‐BP was determined by the addition of CHX in the presence of lysosomal inhibitors (Baf A1, NH4Cl, Chloroquine and Leupeptin) and the proteasome inhibitor carfilzomib. The graph on the right shows the changes in the relative residual levels of HDAC8. r) Degradation of HDAC8 in Huh7 cells treated with indicated constructs was determined by the addition of CHX in the presence of lysosomal inhibitors (Baf A1, NH4Cl, chloroquine and leupeptin) and the proteasome inhibitor carfilzomib. The graph on the right shows the changes in the relative residual levels of HDAC8.

ZDHHC12 mediates protein palmitoylation by transferring the palmitoyl CoA that is generated from PA to the Cys residues of downstream proteins. However, the downstream candidate proteins are not clear. We performed proteomic analysis (**Figure** **3**a) and mass spectrometry (Figure 3b) to identify proteins that mediate the downstream functions of ZDHHC12. Among the top 50 proteins with the highest binding potential (ion intensity) to ZDHHC12, only HDAC8 presented upregulated protein levels in the high‐red‐meat diet group (Figure 3c,d). Subsequent experiments confirmed the interaction between ZDHHC12 and HDAC8 (Figure 3e–m and Figure S6b,c, Supporting Information). Our results demonstrated that HDAC8 was upregulated in tumor tissues compared with normal tissues (Figure S6d–f, Supporting Information), that HDAC8 expression was elevated in the high‐protein diet group compared with the normal diet group (Figure S6g–i, Supporting Information), and that increased HDAC8 levels were correlated with a poor prognosis (Figure S6j, Supporting Information). A reanalysis of the merged dataset shown in Figure S2A (Supporting Information) revealed that HDAC8 was predominantly expressed in HCC cells and was expressed at the highest level in Subcluster 0, which exhibited the most aggressive malignant phenotype (Figure S6k,l, Supporting Information). Hence, we hypothesized that ZDHHC12 increases the stability of HDAC8. ZDHHC12 knockdown did not alter the mRNA level of HDAC8 but decreased its protein level (Figure 3n,o). Tissue microarray staining revealed that the ZDHHC12 and HDAC8 protein expression levels were positively correlated (Figure 3p). Our subsequent experiments revealed that the degradation of HDAC8 was accelerated by the addition of the palmitoylation inhibitor 2‐BP or by the knockdown of ZDHHC12 (Figure 3q,r and Figure S3a–d, Supporting Information). Furthermore, lysosomal inhibitors, such as Baf‐A1 and NH_4_Cl, reversed this degradation effect, but the proteasome inhibitor carfilzomib did not (Figure 3q,r and Figure S7a–d, Supporting Information). However, whether HDAC8 is involved in PA‐associated HCC progression remains unknown. The promotional effect of HDAC8 on HCC has been well studied. HDAC8 promotes β‐catenin activation in NAFLD‐associated HCC^[^ ^22^ ^]^ and promotes HCC cell proliferation by regulating glycolysis and the nuclear localization of PKM2.^[^ ^23^ ^]^ In addition, HDAC8 promotes tumor immune escape in HCC.^[^ ^24^ ^]^ We further confirmed that HDAC8 promotes HCC progression through previously reported oncogenic pathways (Figure S7e, Supporting Information), which is consistent with prior findings.^[^ ^22^ ^]^ Our subsequent results demonstrated that PA upregulates HDAC8 via ZDHHC12 (Figure S7f, Supporting Information) and that PA promotes HCC cell proliferation in an HDAC8‐dependent manner (Figure S7g,h, Supporting Information). In conclusion, our results suggest that HDAC8 binds to ZDHHC12 and may be a key downstream protein.

### 2.4 ZDHHC12 Catalyzes the Palmitoylation of HDAC8 at Cys244

**Figure 4:**
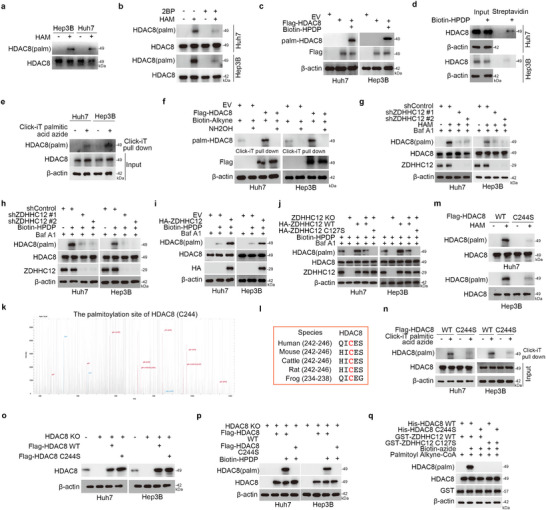
ZDHHC12 catalyzes the palmitoylation of HDAC8 at Cys244. a) In Hep3B and Huh7 cells, HDAC8 was immunoprecipitated using an anti‐HDAC8 antibody, followed by the acyl‐biotin exchange (ABE) assay performed either in the presence or absence of hydroxylamine (HAM) treatment, with subsequent streptavidin‐HRP pulldown of biotin‐conjugated proteins to specifically detect palmitoylated HDAC8. b) In Hep3B and Huh7 cells, HDAC8 was immunoprecipitated using an anti‐HDAC8 antibody, followed by the acyl‐biotin exchange (ABE) assay performed with or without hydroxylamine (HAM) treatment, and subsequently enriched with streptavidin‐HRP to isolate biotin‐conjugated proteins, thereby enabling the detection of HDAC8 palmitoylation levels in both 2‐BP (25 µm, 24 h)‐treated and untreated conditions. c) Huh7 and Hep3B cells were transfected with indicated constructs for 24 h. ABE assay was used to detect the palmitoylation level of HDAC8. d) Huh7 and Hep3B cells were collected for ABE assay to detect the palmitoylation level of HDAC8. e) Huh7 and Hep3B cells treated with or without palmitic acid azide were collected for Click‐IT reaction and streptavidin pulldown. f) Huh7 and Hep3B cells were transfected with indicated constructs for 24 h and then were treated with 100 mm palmitic acid azide for 6 h. The proteins were reacted with biotin‐alkyne and enriched with streptavidin beads after treatment with or without hydroxylamine. g) Huh7 and Hep3B cells were transfected with indicated constructs for 72 h. Cells were treated with Baf A1 for 6 h. ABE assay was used to detect the palmitoylation level of HDAC8 omitting the HAM processing or not. h) Huh7 and Hep3B cells were transfected with indicated constructs for 72 h. Cells were treated with Baf A1 for 6 h. ABE assay was used to detect the palmitoylation level of HDAC8. i) Huh7 and Hep3B cells were transfected with indicated constructs for 24 h. Cells were treated with Baf A1 for 6 h. ABE assay was used to detect the palmitoylation level of HDAC8. j) Generation of stable ZDHHC12‐knockout Huh7 and Hep3B cell lines. 24 h after transfection with the indicated constructs, cells were treated with Baf A1 for 6 h. The ABE assay was employed to assess HDAC8 palmitoylation levels. kl) Palmitoylated peptides mapping of HDAC8 and cross‐species consensus sequence. m) Huh7 and Hep3B cells were collected and ABE assay was used to detect the palmitoylation level of HDAC8 omitting the HAM processing or not. n) Huh7 and Hep3B cells were transfected indicated constructs for 24 h, and then were treated with or without palmitic acid azide. Cells were collected for Click‐iT reaction and streptavidin pulldown. o) Generation of stable ZDHHC12‐knockout Huh7 and Hep3B cells. They were transfected with indicated constructs for 24 h, and then were collected for western blot. p) Generation of stable ZDHHC12‐knockout Huh7 and Hep3B cells. They were transfected indicated constructs for 24 h. ABE assay was used to detect the palmitoylation level of HDAC8. q) GST‐ZDHHC12 or GST‐ZDHHC12 C127S mutant plasmids were transfected into 293T cells. Following cell lysis, the lysates were incubated with glutathione‐agarose beads, and the bead eluates were subsequently mixed with purified His‐HDAC8 or His‐HDAC8 C244S proteins in a reaction buffer containing Palmitoyl Alkyne‐CoA. The Click‐iT reaction was then performed using biotin‐azide, followed by streptavidin‐mediated precipitation of the complexes. Finally, palmitoylated HDAC8 was analyzed by Western blotting after elution.

We further explored whether HDAC8 is palmitoylated by ZDHHC12. The results of ABE and click chemistry experiments indicated that the palmitoylation of HDAC8 occurred (**Figure** **4**a–f). ZDHHC12 increased the palmitoylation level of HDAC8 (Figure 4g–i). Knockout of ZDHHC12 abolished detectable levels of palmitoylated HDAC8, while overexpression of wild‐type ZDHHC12 in the knockout background restored HDAC8 palmitoylation (Figure 4j). In contrast, overexpression of a catalytically inactive ZDHHC12 mutant failed to rescue this effect (Figure 4j). We further searched for the palmitoylation site of HDAC8. We detected the palmitoylation modification of HDAC8 at Cys244 by palmitoylation proteomics, and the cysteine residue at this site is conserved across species (Figure 4k,l). Compared with that of WT HDAC8, the overexpression of HDAC8 with a mutated palmitoylation modification site (C244S) resulted in a significantly lower palmitoylation level of HDAC8 (Figure 4m,n). Our initial experiments successfully confirmed the knockout efficiency of HDAC8 (Figure 4o). In the context of HDAC8 knockout, the overexpression of WT HDAC8 led to the detection of palmitoylated HDAC8, whereas the overexpression of the HDAC8 C244S mutant did not (Figure 4p). As shown, HDAC8 was palmitoylated only when His‐HDAC8 WT, GST‐ZDHHC12 WT, palmitoyl alkyne and biotin‐azide were all present (Figure 4q). However, the absence of palmitoyl alkyne or biotin‐azide, the HDAC8 C244S mutation (which prevents HDAC8 palmitoylation), or the ZDHHC12 C127S mutation (which abolishes ZDHHC12's palmitoyltransferase activity) each resulted in undetectable HDAC8 palmitoylation (Figure 4q). In conclusion, our results suggest that ZDHHC12 catalyzes the palmitoylation of HDAC8 at Cys244.

### 2.5 HDAC8 Palmitoylation at Cys244 Inhibits Its Degradation via the Lysosomal Pathway

**Figure 5:**
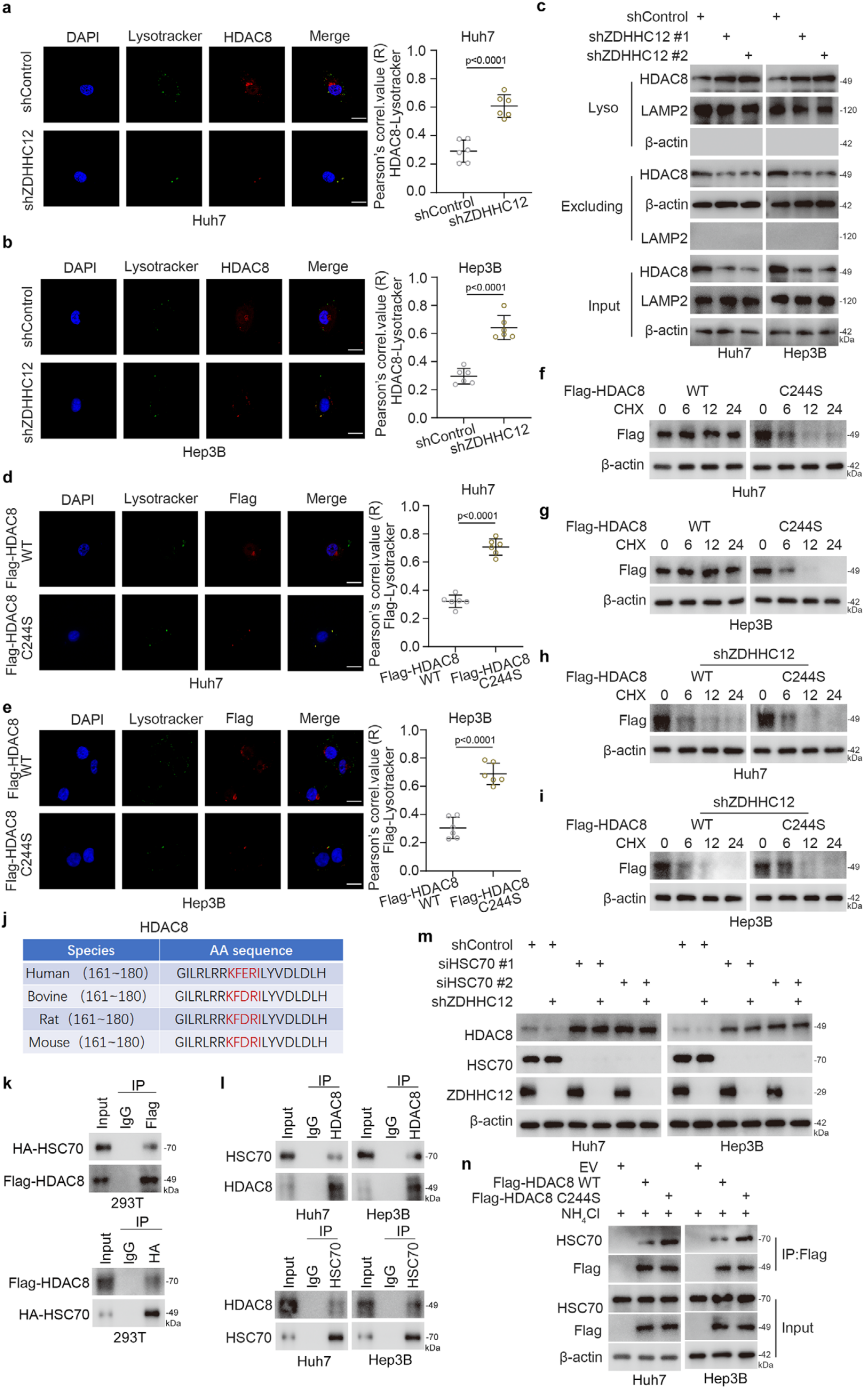
HDAC8 palmitoylation at Cys244 inhibits its degradation via the lysosomal pathway. a,b) representative images of HDAC8, Lysotracker and DAPI immunofluorescence staining in a) Huh7 and b) Hep3B cells with or without ZDHHC12 knockdown. Scale bar: 7.5 µm; *n* = 6 biologically independent experiments, two‐tailed unpaired t test. c) Huh7 and Hep3B cells were transfected indicated constructs for 72 h. Cells were collected and lysosomal and extra‐lysosomal protein components were isolated for western blot analysis. d,e) Representative images of HDAC8, Lysotracker and DAPI immunofluorescence staining in d) Huh7 and e) Hep3B cells with Flag‐HDAC8 WT or Flag‐HDAC8 C244S overexpression. Scale bar: 7.5 µm; *n* = 6 biologically independent experiments, two‐tailed unpaired t test. f) Huh7 and g) Hep3B cells were transfected indicated constructs for 24 h and then were treated with CHX. Cells were collected for western blot analysis. h) Huh7 and i) Hep3B cells were transfected indicated constructs for 48 hours and then were treated with CHX. Cells were collected for western blot analysis. j) Cross‐species stability sequence of HDAC8. k) 293T cells were transfected with indicated constructs for 24 h and collected for immunoprecipitation assay. l) Huh7 and Hep3B cells were collected for immunoprecipitation assay. m) Huh7 and Hep3B cells were transfected with indicated constructs for 72 h. Cells were collected for immunoprecipitation assay and western blot analysis. n) Huh7 and Hep3B cells were transfected with indicated constructs for 24 h and treated with NH4Cl for 8 h. Cells were collected and subjected to immunoprecipitation using anti‐Flag antibody followed by Western blot analysis to examine protein interactions.

We found that the inhibition of palmitoylation promoted HDAC8 degradation and that this process could be reversed by lysosomal inhibitors (Figures 3q,r and S7a—d, Supporting Information). We therefore explored whether the palmitoylation of HDAC8 at Cys244 by ZDHHC12 affects HDAC8 stability. First, we found that HCC cell proliferative capacity remained unchanged upon HDAC8 knockdown, regardless of whether ZDHHC12 was simultaneously knocked down or not (Figure S8a,b, Supporting Information). ZDHHC12 knockdown in HCC cells increased the colocalization of the HDAC8 protein with lysosomal markers (**Figures** **5**a,b and S8c,d, Supporting Information). ZDHHC12 knockdown in HCC cells also decreased the protein levels of HDAC8 in whole‐cell lysates and cellular components other than lysosomes but increased the protein level of HDAC8 in lysosomes (Figure 5c). Compared to HDAC8 WT, the tumor‐promoting effect of the HDAC8 C244S mutant was abolished in HCC proliferation (Figure S9a—f, Supporting Information). Compared with HDAC8 WT, the overexpressed HDAC8 C244S mutant showed increased colocalization with lysosomal markers and lost its stability (Figures 5d,e and S9g—i, Supporting Information). In the case of ZDHHC12 knockdown, there was no difference in protein stability between WT HDAC8 and the HDAC8 C244S mutant (Figure 5h,i). Thus, the palmitoylation of HDAC8 inhibits its degradation via the lysosomal pathway. Analysis of the HDAC8 amino acid sequence revealed a KFERQ‐like sequence, which is conserved among species (Figure 5j). HSC70 is a molecular chaperone that recognizes soluble cytoplasmic proteins with KFERQ‐like motifs and mediates the translocation of its substrates into lysosomes for luminal degradation.^[^ ^25^ ^]^ In a previous study, we reported that HSC70 recognized the KFERQ‐like sequence of YTHDF3 for YTHDF3 degradation.^[^ ^26^ ^]^ Therefore, we hypothesized that the lysosomal degradation of HDAC8 is accomplished after this KFERQ‐like sequence is recognized. Our immunoprecipitation results indicated that HSC70 interacted with HDAC8 (Figure 5k,l). ZDHHC12 knockdown in hepatocellular carcinoma cells decreased the level of HDAC8 expression, but when HSC70 or LAMP2A was knocked down, HDAC8 was not degraded, with or without ZDHHC12 (Figures 5m and S9j, Supporting Information). In addition, compared with WT HDAC8, the overexpressed HDAC8 C244S mutant showed increased binding to HSC70 (Figure 5n). Thus, our results suggest that ZDHHC12‐mediated palmitoylation of HDAC8 at Cys244 inhibits its degradation via the autophagic–lysosomal pathway.

### 2.6 Targeting HDAC8 Could Be a Therapeutic Option for HCC That Is Due to a High‐PA Diet

**Figure 6:**
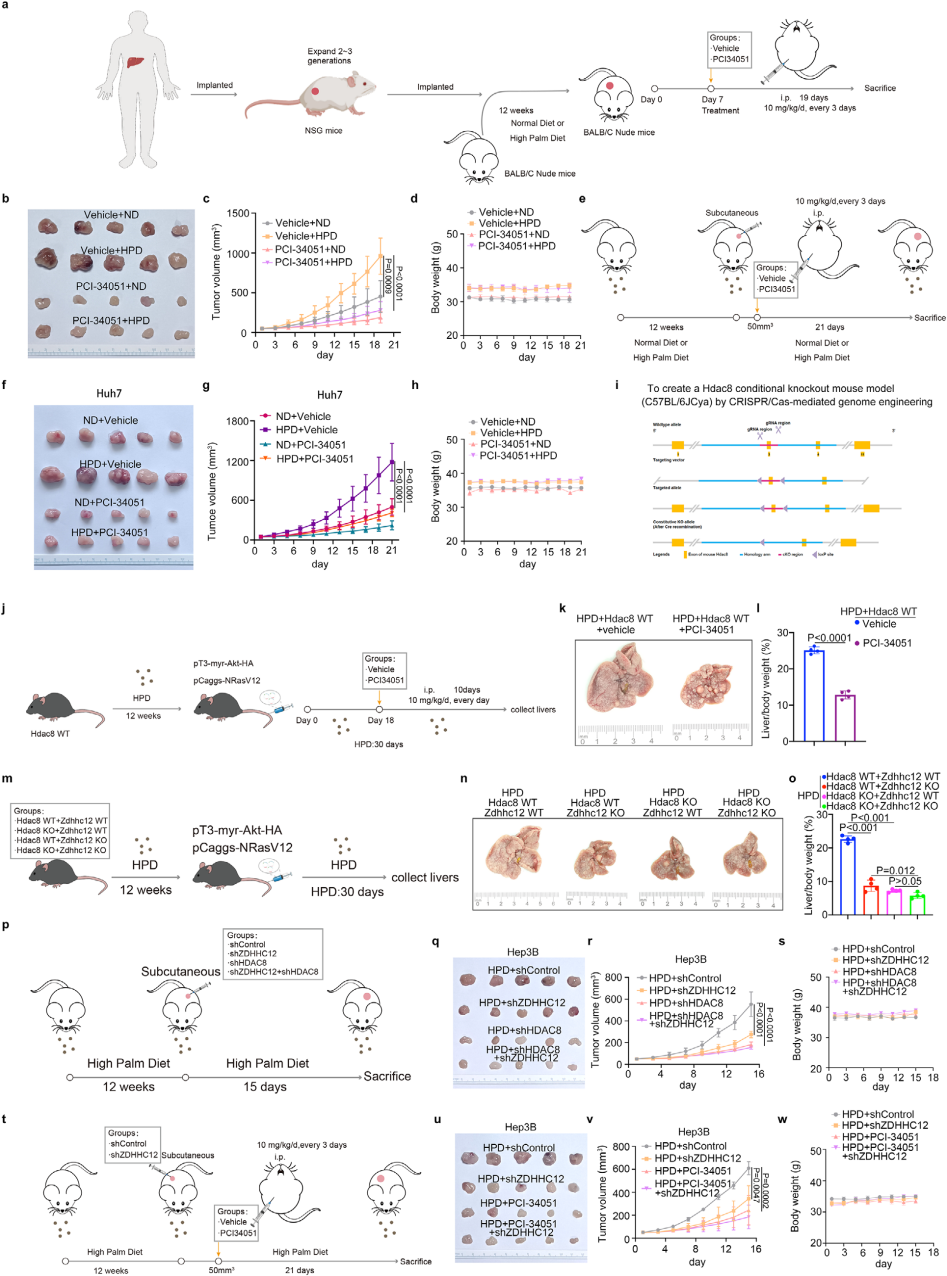
Targeting HDAC8 could be a therapeutic option for HCC that is due to a high‐PA diet. a–d) Tumor tissues from hepatocellular carcinoma patients on a high saturated fatty acid diet were used to construct a PDX model. The tumor‐bearing nude mice were treated with vehicle or PCI‐34051 (10 mg/kg/day, every 3 d). a) Schematic representation of the PDX model construction and PCI‐34051 treatment. b) The photograph of removed tumors. c) The growth curves of the tumors. d) Changes in body weight of treated mice. e) Schematic representation of Huh7 CDX models fed a high palmitic acid diet or a normal diet with or without PCI‐34051 treatment. f) Photographs of tumors, g) growth curves and h) body weight changes of mice in different treatment groups. i) Schematic explanation of the gene targeting strategy. The *Hdac8* to be knocked out is flanked by two loxP sites in the same orientation (loxP‐Hdac8‐loxP). This Flox mouse was mated with Cre mice to obtain offspring mice with *Hdac8* knocked out in the liver by Cre recombinase. j–l) Liver cancer was induced by tail vein injection of pT3‐myr‐Akt‐HA and pCaggs‐NRasV12 plasmids in *Hdac8* WT mice fed with high palmitic acids diet (HPD) for 12 weeks. This was followed by treatment of vehicle or PCI‐34051 (10 mg/kg/d, i.p., per day) for j) 10 d. k) Mouse livers were then collected and photographed, l) subjected to calculation of liver weight ratios. m) Liver cancer was induced in *Hdac8* WT/KO or *Zdhhc12* WT/KO mice fed with high palmitic acids diet (HPD) for 12 weeks. n) Mouse livers were then collected and photographed, o) subjected to calculation of liver weight ratios. p–s) Hep3B cells were transfected with indicated constructs. Puromycin selection was initiated 72 h post‐transfection. p) After successful selection, stably transfected cells were collected and subcutaneously injected into the dorsal flank of nude mice had fed with high palmitic acids diet (HPD) or normal diet (ND) for 12 weeks. q) After 19 d, the tumors were collected and photographed. r) The tumor growth curves and s) body weight curves were drawn. t–w) Hep3B cells were transfected with indicated constructs. Puromycin selection was initiated 72 h post‐transfection. After successful selection, stably transfected cells were collected and subcutaneously injected into the dorsal flank of nude mice that had been fed a high palmitic acid diet (HPD) for 12 weeks. t) When tumors reached 50 mm^3^, mice were injected with PCI‐34051 (10 mg/kg/d, i.p.) every 3 d. u) After 19 d, the tumors were collected and photographed. v) The tumor growth curves and w) body weight curves were drawn.

ZDHHC12 is essential for HPD‐driven HCC progression. However, given the current lack of ZDHHC12 inhibitors and the functional dependence of ZDHHC12 on palmitoylation of downstream proteins, we sought to block its function by targeting key downstream effectors. PCI‐34051, an inhibitor of HDAC8, has been shown to have antitumor effects in previous studies.^[^ ^27^ ^]^ HDAC8 is a key downstream protein of PA that promotes HCC progression; therefore, we verified whether PCI‐34051 is suitable for the treatment of HCC caused by a high‐PA diet. We first investigated the effects of PCI‐34051 in HCC cell lines. Similar to HDAC8 knockdown, PCI‐34051 inhibited the activation of known HDAC8 downstream pro‐oncogenic pathways (Figure S10a, Supporting Information). Notably, in the context of HDAC8 knockdown, PCI‐34051 failed to further suppress these downstream pathways (Figure S10b, Supporting Information) or additionally inhibit HCC progression (Figure S10c, Supporting Information). The PDX and CDX models revealed that a high‐PA diet promoted HCC progression, but after treatment with the HDAC8 inhibitor PCI‐34051, the tumors shrank significantly, and the extent of shrinkage was greater than that in the normal diet‐fed group (**Figures** **6**a–h and S10d—g, Supporting Information). In addition, in vivo experiments revealed that PCI‐34051 treatment was effective in an HCC model fed a high‐PA diet (Figure 6j–l and Figure S11a, Supporting Information). We constructed genetically engineered *Hdac8* knockout mice (Figure 6i and Figure S11b—d, Supporting Information). The results in the HCC model showed that HCC growth was significantly inhibited in high‐PA diet‐fed mice after *Hdac8* was knocked down, regardless of whether *Zdhhc12* was knocked down or not (Figure 6m‒o and Figure S12a, Supporting Information). Similarly, CDX model intervention results indicated that the knockdown of HDAC8 significantly inhibited hepatocellular carcinoma growth in high‐palmitate diet‐fed mice, with or without the knockdown of ZDHHC12 (Figure 6p–w and Figure S12b—e, Supporting Information). Finally, in vivo experiments using the CDX model demonstrated HDAC8 overexpression promotes HCC progression, whereas overexpression of the HDAC8 C244S mutant showed negligible enhancement of HCC proliferative capacity (Figure S13a—d, Supporting Information). In conclusion, our results suggest that targeting HDAC8 could be a therapeutic option for HCC that is due to a high‐PA diet.

**Figure 7:**
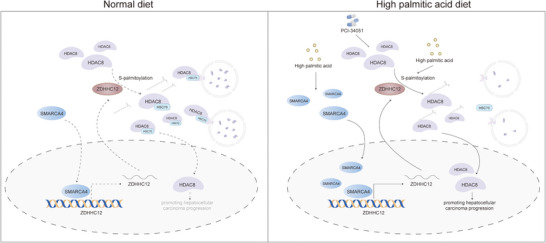
Schematic diagram. PA upregulates ZDHHC12 via SMARCA4 and participates in the palmitoylation of HDAC8 by ZDHHC12. ZDHHC12 inhibits the lysosomal degradation of HDAC8 by palmitoylating the Cys244 of HDAC8, which ultimately promotes the progression of HCC. Targeting HDAC8 may inhibit this process.

## 3 Discussion

SFAs are widely recognized as significant risk factors for metabolic and cardiovascular diseases because of their structural stability.^[^ ^28^ ^]^ Currently, research on SFAs has focused primarily on their effects on cardiovascular disease. Excessive SFA intake accelerates hepatic cholesterol synthesis, leading to elevated cholesterol levels.^[^ ^29^ ^]^ Excess cholesterol accumulation in arterial walls increases cardiovascular disease risk.^[^ ^30^ ^]^ Furthermore, SFAs impair insulin signaling pathways, reduce cellular insulin sensitivity, and disrupt glucose metabolism, potentially contributing to hyperglycemia and diabetes.^[^ ^31^ ^]^ In the context of oncology research, while most studies have shown that SFAs have protumorigenic effects, there are some reports of environmentally dependent anticancer effects of SFAs. Recent clinical studies have further established a close association between SFA‐induced dysregulation of lipid metabolism and HCC.^[^ ^32^ ^]^ However, the role of SFAs in HCC and their mechanisms still need to be further explored. Because of the large number of SFAs, we chose PA, the most common SFA, for a more detailed study. In this study, we demonstrated for the first time that PA promotes HCC progression via ZDHHC12‐mediated palmitoylation of HDAC8 at the Cys244 residue. Although our study, similar to several other studies, suggested that PA can promote cancer progression, there are different points of view.^[^ ^10^, ^13^, ^33^ ^]^ For example, PA has been shown to induce cancer cell apoptosis through mitochondrial pathways and to exert antitumor effects via reactive oxygen species (ROS) generation.^[^ ^34^ ^]^ PA also induces G1‐phase cell cycle arrest and suppresses the PI3K/Akt signaling pathway to inhibit prostate cancer proliferation and metastasis.^[^ ^35^ ^]^ These inconsistencies may be due to the different types of cancers and the different roles of PA‐regulated molecules in different cancers. These findings highlight the complex functional duality of PA in cancer biology and indicate the need for further mechanistic investigations.

Currently, the role of ZDHHC12 in cancer remains poorly understood. ZDHHC12 promotes ovarian cancer progression and cisplatin resistance.^[^ ^36^ ^]^ In addition, ZDHHC12 is thought to promote glioma progression.^[^ ^37^ ^]^ The palmitoylation of NLRP3 by ZDHHC12 inhibits the inflammatory response, and although there are no relevant studies in cancers, this finding suggest that ZDHHC12 inhibits cancer progression.^[^ ^17^, ^38^ ^]^ The role of ZDHHC12 in HCC remains underexplored. This study reveals a novel mechanism whereby ZDHHC12, under high‐SFA dietary conditions, catalyzes HDAC8 palmitoylation at Cys244 to inhibit its autophagic–lysosomal degradation, thereby promoting HCC progression. Unlike previous studies, our work focused on specific dietary factors and employed multiple in vivo models to characterize the function of ZDHHC12 systematically, establishing its critical role in hepatocarcinogenesis and the therapeutic potential of HDAC8 inhibitors for HCC.

HDAC8, a Zn^2^⁺‐dependent class I deacetylase, plays essential roles in cell cycle regulation, gene transcription, and translation. Prior studies have demonstrated the importance of HDAC8 in HCC pathogenesis.^[^ ^23^, ^39^ ^]^ For example, HDAC8 overexpression in HCC cells promotes HCC cell proliferation and survival by altering chromatin structure and gene expression. Notably, HDAC8 promotes HCC progression by promoting HCC cell proliferation and facilitating HCC immune escape.^[^ ^22^, ^23^, ^24^ ^]^ This study bridges the pathological link between high‐SFA diets and HCC by elucidating a novel molecular mechanism. Specifically, ZDHHC12‐mediated HDAC8 palmitoylation stabilizes HDAC8 and suppresses its lysosomal degradation to drive HCC progression. Experimental validation revealed that the HDAC8‐selective inhibitor PCI‐34051 effectively blocked this regulatory axis, providing a rationale for HDAC8‐targeted therapies.

However, this study has several limitations. First, there are multiple types of SFAs, and we chose PA, which is the most common SFA, as an example for this study. The mechanisms of action of other types of SFAs still need to be explored. Second, although there are other downstream targets of ZDHHC12, this study investigated mainly HDAC8 and did not provide a comprehensive understanding of the ZDHHC12 functional network. Third, owing to its specificity limitation, PCI‐34051, the HDAC8 inhibitor that was used in this study, may introduce off‐target effects, which complicate data interpretation and create uncertainty for clinical translation. Fourth, HDAC8 may lose its oncogenic effects in specific cancer types, such as uroepithelial carcinoma, in which its procarcinogenic effects are limited.^[^ ^40^ ^]^ Thus, for cancer patients on a high‐PA diet, it may be necessary to consider the cancer type before selecting PCI‐34051 as the treatment method. This needs to be explored in future studies.

In summary, MR analysis revealed a causal relationship between SFA intake and HCC risk. Follow‐up experiment were conducted with PA, the most common SFA. We identified ZDHHC12 as a key protein for PA‐induced HCC. We found that PA upregulates ZDHHC12 by activating SMARCA4. ZDHHC12 subsequently inhibits HSC70‐mediated lysosomal degradation of HDAC8 by palmitoylating HDAC8 at Cys244, which ultimately promotes HCC progression. Finally, we found that targeting HDAC8 inhibited PA‐induced HCC progression. Our study provides a reference for the personalized treatment of HCC patients on high‐SFA diets, especially high‐PA diets (Figure 7).

## 4 Experimental Section

### Cell Line and Cell Culture

This study utilized the human liver cancer cell lines Huh‐7 (Cat# CL‐0120, Procell, China), Hep3B (Cat# CL‐0102, Procell, China), and 293T (Cat# CL‐0005, Procell, China). All cell lines underwent rigorous short tandem repeat (STR) profiling to ensure authenticity. The cells were cultivated in a humidified incubator at 37 °C with 5% CO2. Huh‐7 and 293T cells were cultured in Dulbecco's modified Eagle medium (DMEM) supplemented with 10% fetal bovine serum (FBS) and 1% penicillin‐streptomycin. Hep3B were maintained in minimum essential medium (MEM) containing 10% FBS and 1% penicillin‐streptomycin. All media and serum were sourced from Gibco (DMEM: #11320033, MEM: #11095080, serum: #A5256701, USA), while penicillin‐streptomycin was obtained from Thermo Fisher Scientific (#15140122, USA).

### Patient Specimens and Criteria for High Red Meat Diet

Patients with liver cancer were enrolled, approved by the Medical Committee of Sichuan Provincial People's Hospital of University of Electronic Science and Technology of China. During surgery (Approval No. 2023526‐1), liver cancer tissues (at least 5 cm from the tumor margin) were collected from all patients. Pathology experts conducted a double‐blind evaluation to confirm the diagnosis. All patients provided informed consent. Patients’ information is summarized in Table S1 (Supporting Information). The WHO International Agency for Research on Cancer (IARC) classifies red meat intake of more than 100 grams per day (about 700 g per week) as “probably carcinogenic” (category 2A).^[^ ^41^ ^]^ Intake exceeding this threshold as a “high red meat diet,” was defined assessed through three consecutive months of patient interviews and evaluation by professional nutritionists. The patients numbered 1–15 in Table S1 (Supporting Information) belong to the normal diet group, while those numbered 16–30 are categorized under the high‐red‐meat diet group. A comprehensive comparative analysis of baseline characteristics between the two groups among 30 hepatocellular carcinoma patients is presented in Tables S2 and S3 (Supporting Information).

### Mice Xenograft Model and High Palmitic Acid Diet

For the CDX (cell line‐derived Xenograft) model, six‐week‐old male nude mice obtained from Vital River were utilized. The mice were housed in a specific pathogen‐free (SPF) animal facility. Five million (5×10⁶) HCC cells were injected subcutaneously into each mouse. For the PDX (patient‐derived Xenograft) model, severely immunodeficient (NOG) mice and nude mice were obtained from Vital River. The samples were cut from liver cancer patients into small pieces, transplanted them into NSG mice, and then passed them to NSG and nude mice for 2–3 generations. Finally, tumors were transplanted into nude mice for the experiment. The high‐palmitate diet (HPD) used for mouse feeding was purchased from Shulb Company and subsequently modified. Specifically, the cocoa butter content in the D12108C formulation was increased from 17.28% to 30%, while proportionally reducing other components and maintaining constant vitamin and mineral supplementation. All animal procedures were approved by the Ethics Committee of the Second Xiangya Hospital of Central South University (Approval No. 20240632).

### Genetically Engineered Mice and Spontaneous Liver Cancer Model

*Zdhhc12* KO genetically engineered mice were designed by Cyagen Biosciences (Suzhou, China). Hdac8 KO genetically engineered mice were designed by Shulaibao Biotechnology (Wuhan, China). Six‐week‐old male C57BL/6 mice were obtained from Vital River and housed in a specific pathogen‐free (SPF) animal facility. To construct the orthotopic hepatocellular carcinoma model, we prepared a plasmid solution containing pT3‐myr‐Akt‐HA (5 µg), pCaggs‐NRasV12 (25 µg), and Sleeping Beauty transposase plasmid (pCMV‐SB, 4 µg) dissolved in 2 mL of saline. For subsequent injections, 2 mL of this solution was administered per 20 g of mouse body weight. Following a 3 d acclimation period, the entire plasmid solution was injected into the mice via the tail vein within 7 s.

### Chemicals and Reagents

PCI‐34051 (Cat# S2012, Selleck); ICG‐001 (Cat# S2662, Selleck); Palmitic acid (Cat# P5585, Sigma); 2‐BP (Cat# E0120, Selleck); biotin‐HPDP (#SML3797, Merck); streptavidin (Cat# 47503ES03, YEASEN); Click‐iT palmitic acid azide (Cat# C10265, Thermo Fisher Scientific). *E. coli* BL21 (Cat# C600003, Thermo Fisher Scientific). The sequences of all small interfering RNAs (siRNAs), single guide RNAs (sgRNAs) and short hairpin RNAs (shRNAs) were listed in Table S4 (Supporting Information). The siRNAs were obtained from RiboBio (Guangzhou, China). The shRNAs and plasmids were purchased from GeneCopoeia (USA). Primary antibodies used were as follows: rabbit polyclonal anti‐ZDHHC12 (Cat# ab237688, abcam), rabbit polyclonal anti‐SMARCA4 (Cat# 21634‐1‐AP, Proteintech), rabbit polyclonal anti‐SP5 (Cat# DF9081, Affinity), rabbit polyclonal anti‐HDAC8 (Cat# 17548‐1‐AP, Proteintech), mouse monoclonal anti‐HDAC8 (Cat# H00055869‐M07, ThermoFisher), rabbit polyclonal anti‐HA (Cat# 51064‐2‐AP, Proteintech), rabbit polyclonal anti‐Flag (Cat# 20543‐1‐AP, Proteintech), mouse monoclonal anti‐Flag (Cat# 66008‐4‐Ig, Proteintech), rabbit polyclonal anti‐Beta Actin (Cat# 20536‐1‐AP, Proteintech). The reagents used for the immunofluorescence assay were Triton X‐100 (Cat# P0096, Beyotime), Lyso Traker Green (Cat# C1047S, Beyotime), CoraLite647‐conjugated F(ab')2 Fragment Goat Anti‐Rabbit IgG (H+L) (SA00014‐9, Proteintech) and CoraLite488‐conjugated Goat Anti‐Mouse IgG(H+L) (SA00013‐1, Proteintech).

### Transient and Stable Transfection

Cells were cultured in plates and starved for 12 h in Opti‐MEM (#31985070, Gibco, USA). Subsequently, plasmid was combined with 250 µL of Opti‐MEM. Lipofectamine 2000 (#11668019, Thermo Fisher Scientific, USA) was combined with 250 µL of Opti‐MEM. After 5‐minute incubation, the plasmid and Lipofectamine mixture was then mixed and incubated for an additional 20 min. This transfection mixture was added to 3500 µL of Opti‐MEM, and cells were transfected for 7 hours. After transfection, Opti‐MEM was replaced with complete medium. To establish a stable cell line, the target cells were seeded in appropriate culture dishes and incubated in complete medium until reaching 60–80% confluence. Concentrated lentiviral particles (containing pTsin‐Flag/HA‐gene or pLKO.1‐shRNA with a puromycin resistance marker) were mixed with Polybrene at a final concentration of 4–8 µg mL^−1^ and added to the cell culture medium. After the designated transduction period, the virus‐containing medium was aspirated, followed by gentle washing of the cells 1–2 times with pre‐warmed complete medium to remove residual viral particles. Subsequently, the cells were treated with puromycin (2 µg mL^−1^, #A1113803, Thermo Fisher Scientific, USA) for 7–10 d to eliminate non‐transfected cells. The puromycin‐containing medium was replaced every 2–3 d. The surviving cells were expanded and validated for stable knockdown.

### Western Blot Analysis

HCC cells were lysed on ice for 30 min using RIPA lysis buffer (#G2002‐100ML, Servicebio, China), supplemented with 1% protease inhibitor (#P1005, Beyotime, China) and 1% phosphatase inhibitor (#P1045, Beyotime, China). Cell lysates were centrifuged at 12 000 rpm and 4 °C for 10 min, and the supernatant was collected to measure protein concentration using the Micro BCA protein assay kit. After adding loading buffer, the supernatant was boiled at 95 °C for 10 min. Proteins were separated by sodium dodecyl sulfate polyacrylamide gel electrophoresis (SDS‐PAGE) and transferred to polyvinylidene difluoride (PVDF) membranes. Following a 1 h blocking step with skim milk at room temperature, the membranes were incubated with primary antibodies at 4 °C overnight. The next day, the membranes were incubated with secondary antibodies at room temperature for 1 hour, and protein signals were developed using enhanced chemiluminescence (ECL) luminescence solution (#34577, Thermo Fisher Scientific, USA).

### Coimmunoprecipitation (co‐IP)

Cell lysates were prepared in RIPA lysis buffer supplemented with protease and phosphatase inhibitors. After centrifugation, the supernatant was incubated with magnetic beads precoated with specific antibodies at 4 °C overnight. Subsequently, the beads were washed 6 times to remove non‐specifically bound proteins and treated with sample buffer and boiling. Finally, proteins were detected by Western blot.

### Glutathione S‐Transferase (GST) Pull‐Down Assay

To extract GST fusion proteins expressed in the BL21 strain (vector: pET‐GST), the bacteria were first lysed with muramidase and sonicated them. Subsequently, the GST fusion proteins were captured overnight at 4 °C using glutathione‐agarose beads (#16100, Thermo Fisher Scientific, USA). After repeated washing, the captured proteins were mixed with cell lysate of the target protein (lysed on ice for 30 min in Western/IP lysis buffer) and incubated overnight at 4 °C. After multiple washings and boiling, the proteins were separated by sodium dodecyl sulfate polyacrylamide gel electrophoresis (SDS‐PAGE), and the gel was stained and imaged using Coomassie Brilliant Blue staining (#G2059, Servicebio, China) to provide a basis for subsequent analysis.

### Quantitative Real‐Time PCR (RT‐qPCR)

Following the manufacturer's instructions, total RNA from cells using TRIzol reagent (#AG21102, Accurate Biotechnology, China) was extracted. The extracted RNA was then quantified using a NanoDrop2000 spectrophotometer (Thermo Fisher Scientific, USA). Next, transcribed RNA was reversed into cDNA using a reverse transcription kit (#AG11728, Accurate Biology, China). The gene‐specific primers used in the experiment were synthesized by BGI (Beijing), and the sequences are listed in Table S5 (Supporting Information). Finally, the expression level of the target gene using the Evo M‐MLV one‐step RT‐qPCR kit (#AG11732, Accurate Biology, China) was quantitatively analyzed.

### Chromatin Immunoprecipitation (ChIP)‐qPCR Analysis

For ChIP‐qPCR experiments, a commercial chromatin extraction kit (#ab117152, Abcam, USA) and the ChIP kit Magnetic‐One Step (#ab156907, Abcam, USA) were utilized. The specific primer sequences used in ChIP‐qPCR are provided in Table S6 (Supporting Information).

### Dual Luciferase Reporter Gene Assay

Cells were spread in 24‐well cell culture plates at a density of 50 000 per well and incubated in a cell culture incubator for 24 h. Subsequently, a firefly luciferase reporter gene plasmid (pGL3‐Basic, Promega) containing the promoter sequence of the target gene and a Renilla luciferase internal reference plasmid (pRL‐SV40, Promega) containing the constitutive promoter were co‐transfected into the above cells using the liposome transfection method. Forty‐eight hours after transfection, the activities of firefly luciferase and sea kidney luciferase were measured. Luminescence values were recorded using a multifunctional enzyme marker during the experiment. The relative luciferase activity was obtained by dividing the activity value of firefly luciferase by that of sea kidney luciferase.

### Immunohistochemistry

A hepatocellular carcinoma (HCC) tissue microarray chip (#D097LV01) from Zhongke Guanghua (Xi'an) Intelligent Biotechnology Co., Ltd. was used for immunohistochemistry (IHC) staining. The IHC staining kit and corresponding antibodies were employed from Bios Biological Technology Co., Ltd. to stain the tissue chip. Two experienced pathologists independently evaluated the IHC staining results. The staining intensity was categorized into three levels: Grade 1 (weak): little staining at 40* magnification; Grade 2 (moderate): moderate staining at 40* magnification; Grade 3 (strong): strong staining at 40* magnification.

### Acyl‐Biotin Exchange (ABE) Assay

To completely block free thiols, cell lysates were incubated with 20 mm methanethiosulfonate (#23011, Thermo Fisher Scientific, USA) and 1 mm PMSF (#ST507, Beyotime, China) at 50 °C for 30 min. Next, proteins were isolated using acetone precipitation and resuspended in 1 m hydroxylamine (pH 7.4, Sigma‐Aldrich) solution to promote depalmitoylation. The treated proteins were incubated with 0.2 mm biotin‐HPDP (#SML3797, Merck, German) at room temperature for 1 h to biotinylate them. Finally, the biotinylated proteins were purified using streptavidin (#47503ES03, YEASEN, China) and detected by immunoblotting.

### Click‐iT Pull Down

Cells were incubated in medium containing 100 µm Click‐iT palmitic acid azide (#C10265, Thermo Fisher Scientific, USA) for 6 h. Next, the cells were lysed, and total protein was extracted. The protein samples were coupled to biotin‐alkyne using the Click‐iT protein reaction buffer kit (# C10276, Thermo Fisher Scientific, USA). Then, the biotinylated palmitoylated proteins were precipitated with streptavidin and detected by SDS‐PAGE and immunoblotting.

### Cell Counting Kit‐8 (CCK‐8) Assay and Colony Formation Assay

CCK‐8 kit (#C0037, Beyotime, China) was used to perform CCK‐8 assay. Cells were seeded at a density of approximately 10 000 cells per well in a 96‐well plate. After culturing for 24 h, the cells were divided into different treatment groups. CCK‐8 solution was added to each well and incubated for 1 h. The absorbance of each well was measured at a wavelength of 450 nm using a microplate reader to assess cell viability. Cell viability was assessed by measuring the absorbance of each well at 450 nm using an enzyme meter. For clone formation experiments, each group of cells was counted, diluted to the same concentration and planted in equal amounts in six‐well plates. After 12 d of incubation, 4% paraformaldehyde fixation and crystal violet staining were performed and finally the number of colony formation was observed.

### Proximity Ligation Assay (PLA)

Cells were seeded on sterile coverslips (#FCP126, Beyotime, China) in 12‐well plates and cultured in an incubator. After 36 h, the culture medium was removed, the cells were washed with PBS, and fixed and permeabilized. Subsequently, to reduce nonspecific binding, the cells were blocked with Duolink blocking solution. Next, antibodies were incubated at 4 °C overnight. Subsequently, cells were washed twice with PBS containing 5% BSA and incubated in PLA probe mixture for 1 hour. Ligation reaction and amplification reaction were performed in sequence according to the instructions of Duolink in situ detection kit (Sigma‐Aldrich, USA). Finally, cells were rinsed with Duolink In Situ Wash Buffer, mounted, and imaged.Immunofluorescence Assay and Quantification.

### Immunofluorescence Assay and Quantification

The reagents used were described in the section “Chemicals and reagents.” Hepatocellular carcinoma cells were incubated with Lyso – Tracker for 20 min, paraformaldehyde fixed for 15 min, and permeabilized with 0.2% Triton X‐100 for 10 min. Cells were incubated with primary antibody overnight at 4 °C and then with fluorescent secondary antibody for 1 h. Cell nuclei were stained with DAPI for 15 min and then analyzed by confocal microscopy. For colocalization quantification, Pearson's correlation coefficient (R) analysis using ImageJ with the JACoP was performed. Dual‐channel analysis was conducted, with each channel representing either red or green fluorescence signals of the respective molecules.

### RNA Sequencing and Proteomics

For bulk‐RNA sequencing, RNA was extracted, and RNA sequencing analysis was performed by Geneseeq Technology (Beijing, China) using an Illumina HiSeq 2500 instrument (Illumina, San Diego, CA) with three replicates per group. Raw reads were pre‐processed to filter out rRNA reads, sequencing adapters, short reads, and other low‐quality reads. Clean reads were mapped to the human reference genome GRCh38 (hg38) using TopHat v2.1.0 with two mismatches. After genome mapping, Cufflinks v2.1.1 was run with the reference annotation to generate fragment and count values for known gene models. For proteomics, the prepared tissue samples were further analyzed by Shanghai Bioproffle Technology Co., Ltd. Cell samples were lysed with 200 µL of lysis buffer (4% SDS, 100 mm DTT, 150 mm Tris‐HCl pH 8.0). The samples were boiled and further sonicated. Unlysed cell debris was removed by centrifugation at 16000 x *g* for 15 min. The supernatant was collected and quantified by BCA. Subsequently, peptide fragment preparation, LC‐MS analysis, database searching and analysis, and bioinformatics analysis were performed. For MS data acquisition, timsTOF Pro2 (Bruker) was operated in PASEF mode. The full scan was recorded from 100 to 1700 m/z, and the mobility (1/K0) dimension spanned from 0.6 to 1.6 Vs cm^−2^. The MS method was set as follows: ramp time 100ms, accumulation time 2.0 ms, lock duty cycle 100%, capillary voltage 1700 V, drying gas 3 L min^−1^, and drying temperature 180 °C.

### Single‐Cell Data Analysis and Other Bioinformatics Analyses

The Seurat R package was used to conduct a comprehensive analysis of single‐cell transcriptome data. After preprocessing the data, dimensionality reduction and clustering methods were employed to classify cells. The Harmony package was used to mitigate batch effects. The FindVariableFeatures function identified the top 2000 variable genes. To accurately annotate cell types, The SingleR package was utilized. Additionally, copy number variation analysis on single‐cell RNA sequencing data using the inferCNV package was performed to identify potential malignant cells. Differential expression analysis was performed using the Deseq2 package or limma package. Enriched pathways were identified with the Kyoto Encyclopedia of Genes and Genomes (KEGG) using the clustetrProfiler, org.Mm.eg.db and org.Hs.eg.db packages. BEST (https://rookieutopia.hiplot.com.cn/app_direct/BEST/) and TIMER2.0 (http://timer.cistrome.org/) were utilized for TCGA data analysis.

### Mendelian Randomization

To investigate the causal relationship between saturated fatty acids (SFAs) and hepatocellular carcinoma (HCC), two‐sample Mendelian randomization (MR) analysis was performed. MR leverages genetic variants as instrumental variables (IVs) to infer causality while minimizing confounding biases.^[^ ^42^ ^]^ Both exposure (SFA, ID: met‐d‐SFA) and outcome (HCC, ID: DGWAS‐5484) genetic associations were sourced from summary‐level data in the DMRdb repository (http://www.inbirg.com/DMRdb/).^[^ ^43^ ^]^ Only genome‐wide significant (*P* < 5 × 10^−^⁸) and independent (LD *r* ^2^ < 0.001) SNPs were selected. Inverse‐variance weighted (IVW) method was used as the primary MR approach. Results were presented in forest plots and scatter plots to illustrate the causal effect. A *P*‐value < 0.05 was considered statistically significant. Statistical Analysis.

### Statistical analysis

The experimental data were statistically analyzed using GraphPad Prism 7.0 software. For comparisons between different experimental groups, we used Student t test, one‐way analysis of variance or two‐way analysis of variance. All statistical results are expressed as mean ± standard deviation.

## Conflict of Interest

The authors declare no conflict of interest.

## Author Contributions

X.J., Y.L.H., and Y.Q.Z. contributed equally to this work. X.J.: project administration, conceptualization, source; Y.H.: methodology, formal analysis; Y.Z.: methodology, formal analysis; W.S.: formal analysis; R.L.: formal analysis; X.Y.: formal analysis, C.Y.: data curation, resources; Y.Z.: Project administration, resources.

## Ethics Approval and Consent to Participate

The study was approved by the Medical Committee of Sichuan Provincial People's Hospital of University of Electronic Science and Technology of China (Approval No. 2023526‐1) for patients’ samples and the Ethics Committee of the Second Xiangya Hospital of Central South University (Approval No. 20240632) for animals.

## Supporting information



Supporting Information

Supporting Information

## Data Availability

Sequencing data used in this study are available in GEO (GSE290907). Other datasets used in the study are available from the corresponding authors on reasonable request.
