# Supplementary material for: ZDHHC12 Palmitoylates HDAC8 to Promote the Progression of Hepatocellular Carcinoma Associated with a Diet High in Saturated Fatty Acids
Source: Adv Sci (Weinh). 2025 Aug 11;12(40):e05702. doi: 10.1002/advs.202505702 (PMC12561377; doi:10.1002/advs.202505702)
Supplement: Supplementary file 1 — Supporting Information [file ADVS-12-e05702-s002.pdf]

ZDHHC12 palmitoylates HDAC8 to promote the progression of hepatocellular carcinoma associated with a diet high in saturated fatty acids

Xin Jin, Yulong Hong, Yongqi Zhao, Wensheng Shi, Ruilin Liu, Xinyu You, Chong Yang, Yu Zhang

Supplementary figure 1

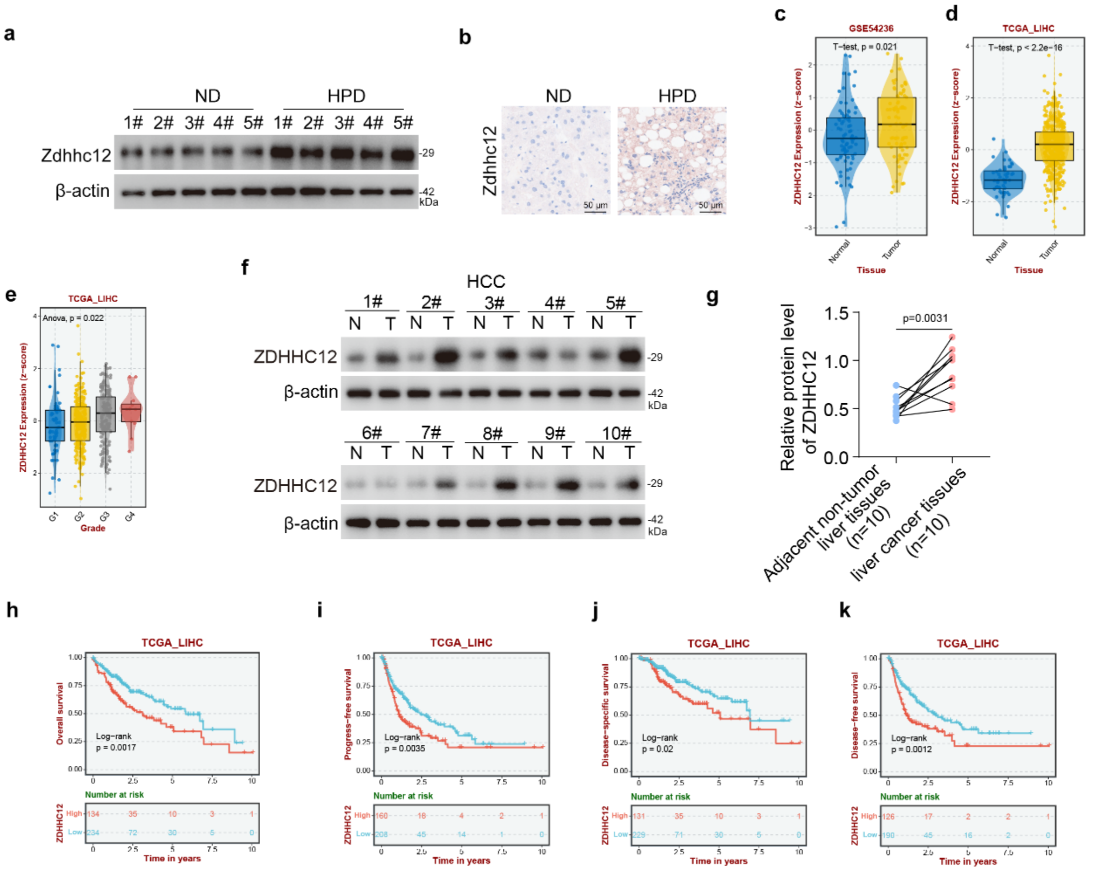

Supplementary figure 1 (related to Figure 1). a and b, liver cancer was induced by tail vein injection of pT3-myr-Akt-HA and pCaggs-NRasV12 plasmids in C57BL/6 mice fed with high palmitic acid diet (HPD) or normal diet (ND) for 12 weeks. Mouse livers were then collected for western blotting (a) and IHC (b). **c-e,** expression levels of ZDHHC12 in specimens from hepatocellular carcinoma patients with different tissue types (c-d) and grades (e). TCGA data analysis was performed using the BEST tool (https://rookieutopia.hiplot.com.cn/app_direct/BEST/).

f and g, ZDHHC12 protein expression levels in cancer tissues and adjacent normal tissues of 10 patients with hepatocellular carcinoma. **h-k**, relationship between ZDHHC12 expression levels and overall survival (OS) (h), progression-free survival (PFS) (i), disease-specific survival (j) and disease-free survival (k) in HCC patients. TCGA data analysis was performed using the BEST tool (https://rookieutopia.hiplot.com.cn/app_direct/BEST/).

Supplementary figure 2

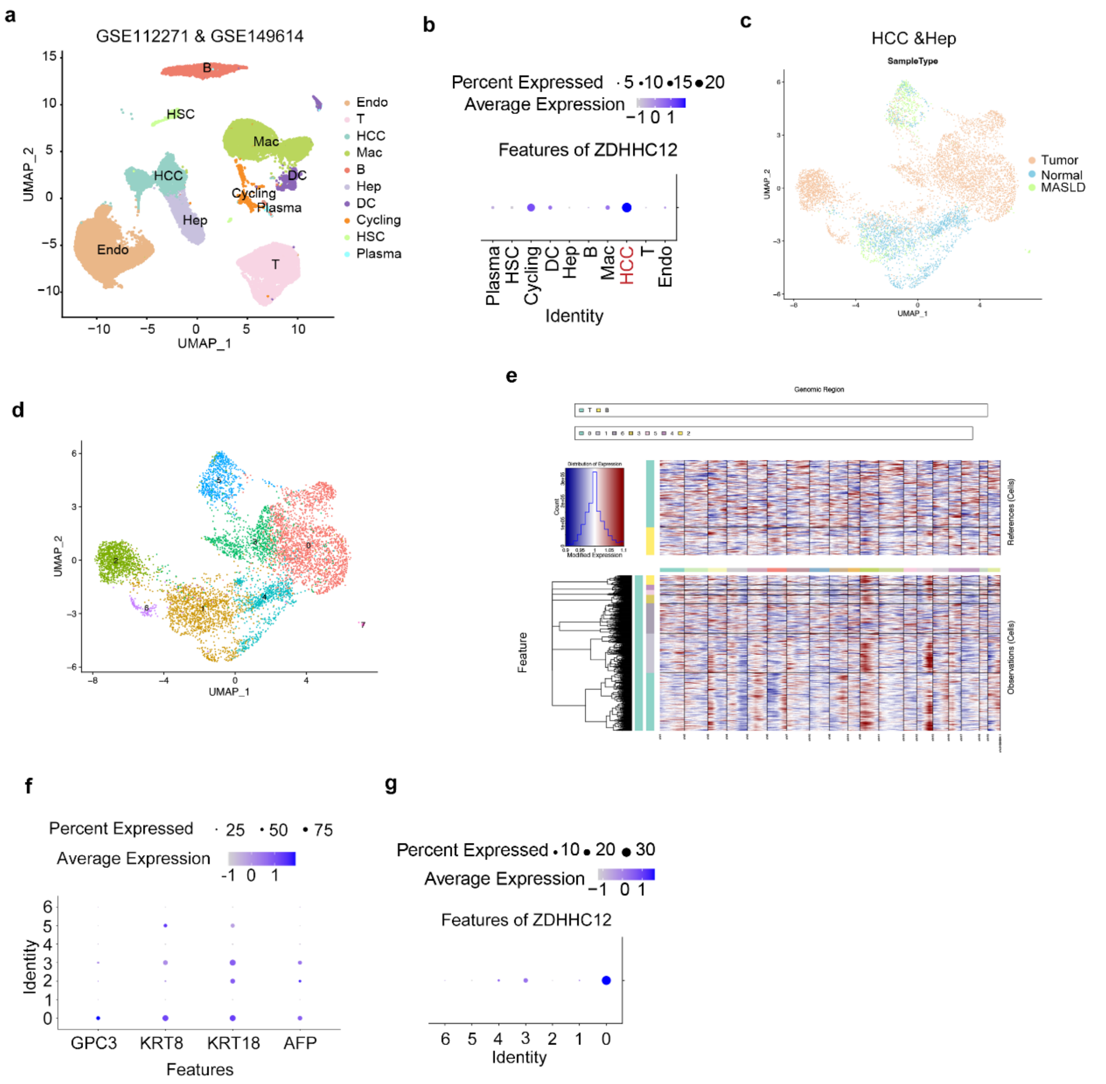

Supplementary figure 2 (related to Figure 1). a, the GSE112271 and GSE149614 datasets were integrated, followed by clustering of all cells and visualization via UMAP plot. **b**, ZDHHC12 expression distribution patterns from two integrated single-cell RNA sequencing datasets (GSE112271 and GSE149614). **c**, the HCC and Hep cell populations were isolated, visualized via UMAP plot, and annotated by their sample origins. **d**, subclustering analysis of extracted epithelial

cell populations. **e and f**, inference of malignant epithelial cells using inferCNV (e) and examination of malignancy marker expression patterns across subclusters (f). **g**, evaluation of ZDHHC12 expression profiles in all subclusters.

Supplementary figure 3

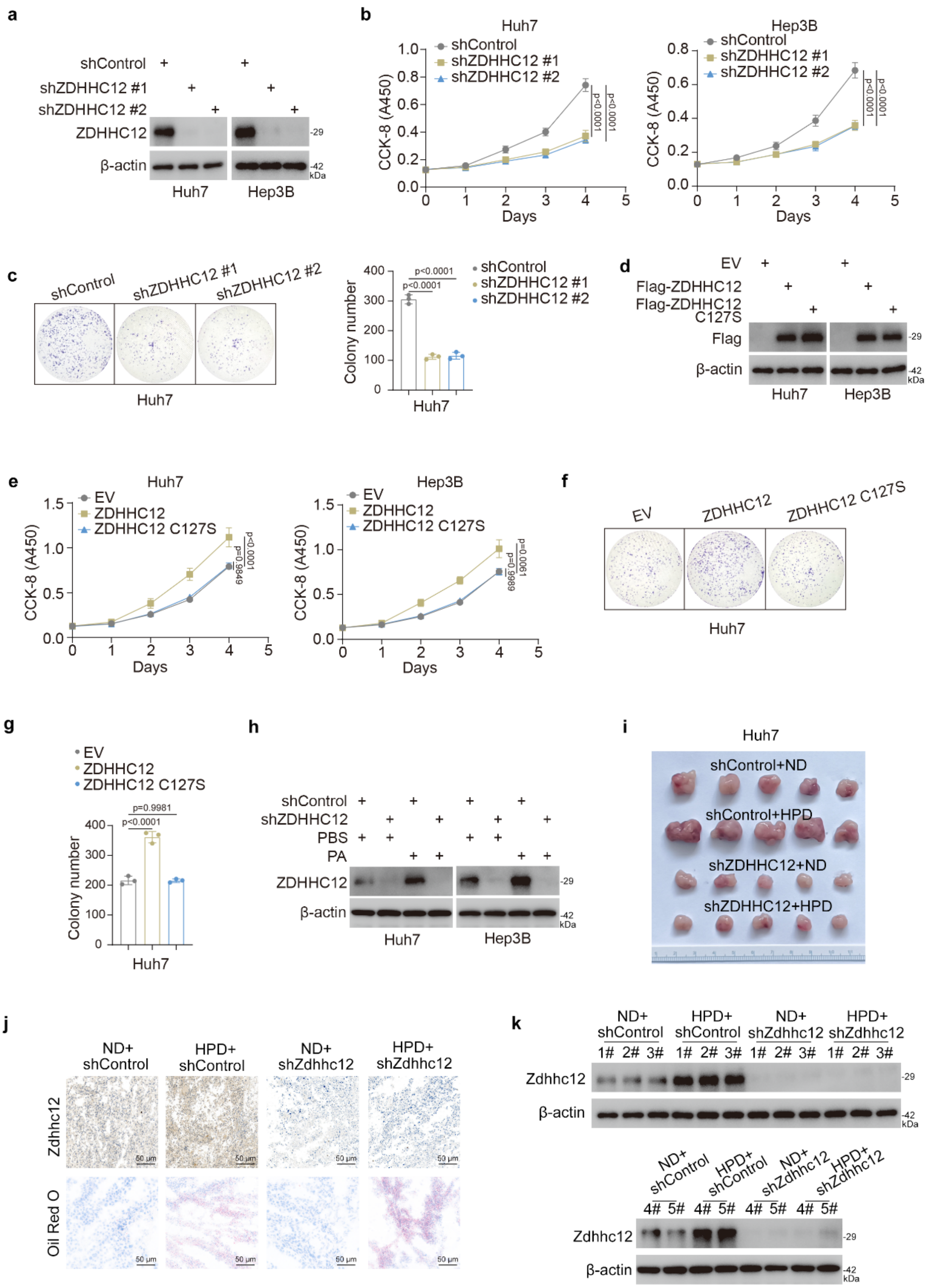

Supplementary figure 3 (related to Figure 1). a-c, cells were transfected with indicated constructs. Puromycin selection was initiated 72 hours post-transfection. After successful selection, stably transfected cells were collected for western blotting (a), CCK-8 assay (b) and colony formation assay (c). **d-g**, cells were transfected with indicated constructs. Puromycin selection was initiated

24 hours post-transfection. After successful selection, stably transfected cells were collected for western blotting (d), CCK-8 assay (e) and colony formation assay (f and g). **h**, Huh7 and Hep3B cells were transfected with shControl or shZDHHC12 plasmids. Puromycin selection was initiated 72 hours post-transfection. After successful selection, stably transfected cells were collected. The cells were treated with PBS or palmitic acid (200 μM) and collected for western blotting. **i-k**, Huh7 cells were transfected with shControl or shZDHHC12. Puromycin selection was initiated 72 hours post-transfection. After successful selection, stably transfected cells were collected and subcutaneously injected into the dorsal flank of nude mice that had been fed a High Palmitic Acid Diet (HPD) or a Normal Diet (ND) for 12 weeks. The tumors were harvested after 21 days, photographed (i), and subjected to IHC, oil o red (j) and Western blotting (k) analysis.

Supplementary figure 4

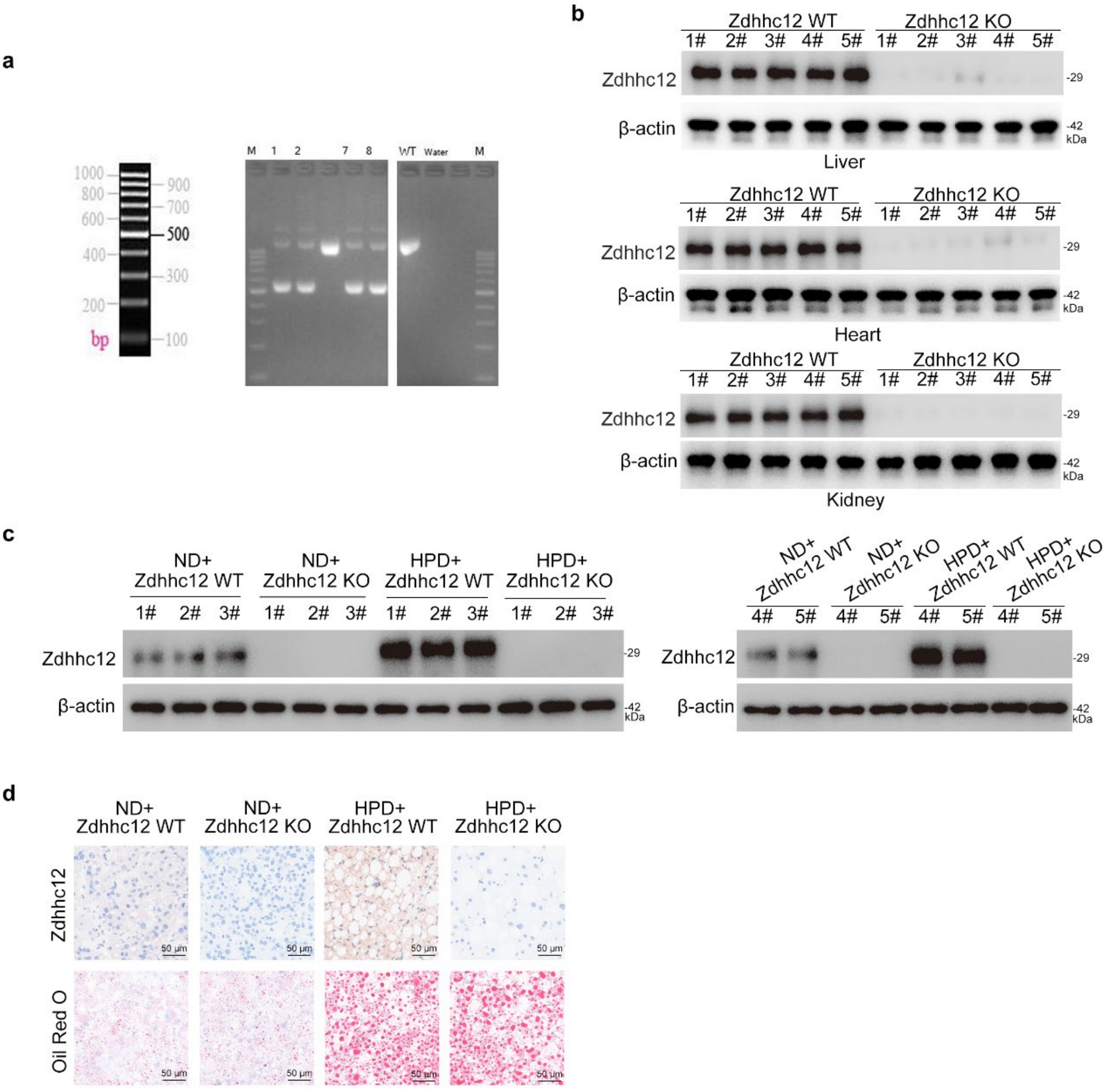

Supplementary figure 4 (related to Figure 1). a, the tail end of the mouse was cut about 0.5 cm, and the tissue was lysed to extract DNA, which was amplified by PCR and then electrophoresed to identify the genetically engineered mice. Our genotyping results confirmed that mice #1, #2, #7, and #8 were positive for the targeted deletion (574-bp band), whereas the lanes between #2 and #7 represented mice negative for the deletion (wild-type or unsuccessful knockout, 1260-bp band), which were excluded from subsequent experiments. **b**, western blot analysis of Zdhhc12 protein expression in various organs of Zdhhc12 wild-type and Zdhhc12 knockout mice. **c**, western blot analysis of Zdhhc12 protein expression in hepatocellular carcinoma tissues of Zdhhc12 wild-type and Zdhhc12 knockout mice hepatocellular carcinoma tumor model undergoing HPD feeding or ND feeding. **d**, IHC and oil o red analysis of Zdhhc12 protein expression in hepatocellular

carcinoma tissues of Zdhhc12 wild-type and Zdhhc12 knockout mice hepatocellular carcinoma tumor model undergoing HPD feeding or ND feeding.

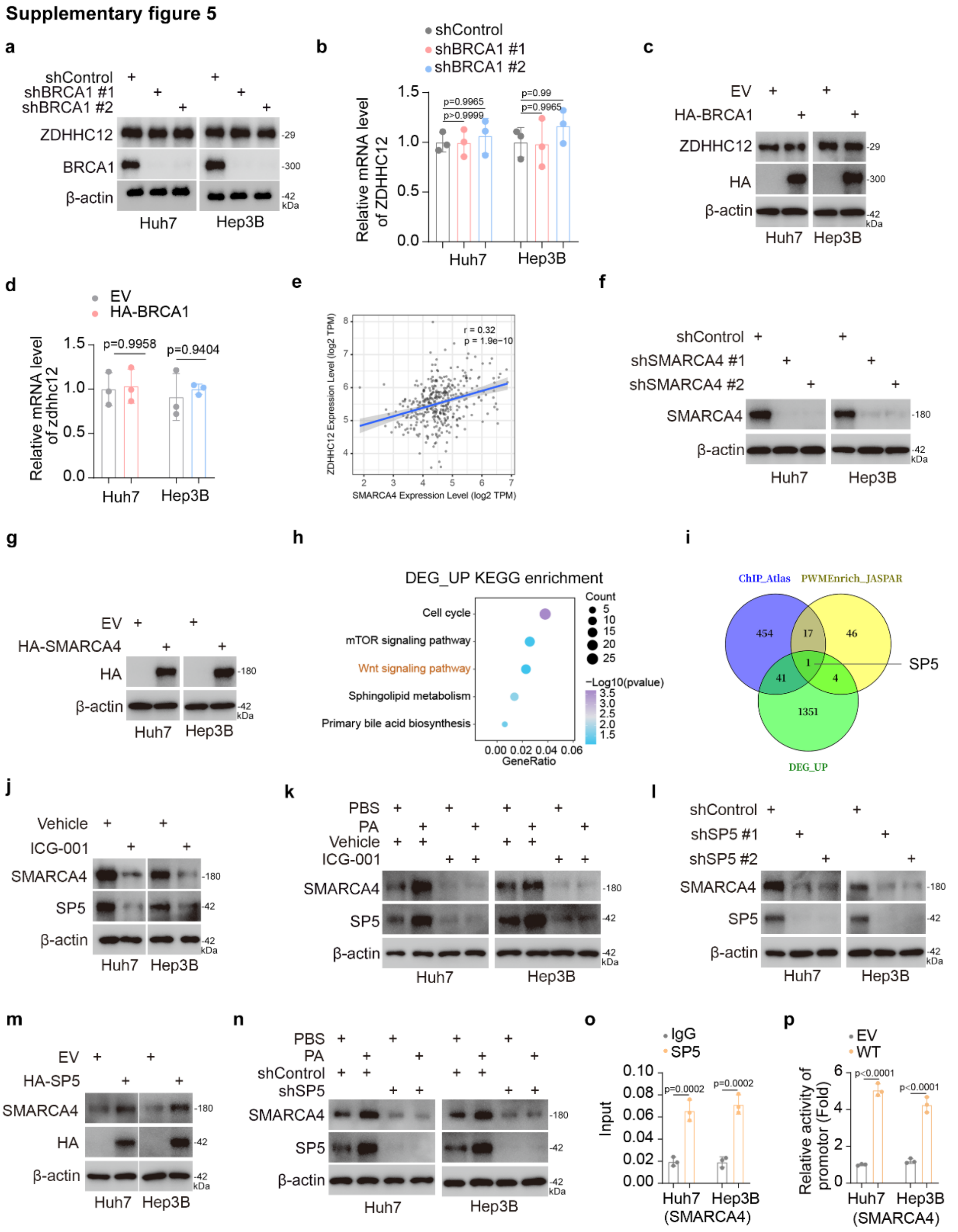

Supplementary figure 5 (related to figure 5). a and b, cells were transfected with indicated constructs for 72 hours and then were collected for western blot analysis (a) and RT-qPCR analysis (b). **c and d,** cells were transfected with indicated constructs for 24 hours and then were collected

for western blot analysis (c) and RT-qPCR analysis (d). **e**, the analysis was performed using TIMER2.0 (http://timer.cistrome.org/) to examine the correlation between ZDHHC12 and SMARCA4 expression levels in TCGA-LIHC data. **f**, cells were transfected with indicated constructs for 72 hours and then were collected for western blot analysis. **g**, cells were transfected with indicated constructs for 24 hours and then were collected for western blot analysis. **h**, analysis of pathways upregulated in the high-red-meat diet group compared to the normal diet group revealed significant activation of the Wnt signaling pathway, among others. **i**, by intersecting potential SMARCA4 transcription factors predicted by ChIP-atlas and JASPAR databases with genes upregulated in the high-red-meat diet group, we identified SP5 as a putative transcriptional regulator of SMARCA4. **j**, cells were treated with vehicle or ICG-001 (10 μM) for 24 hours and collected for western blotting analysis. **k**, cells were treated with either vehicle or ICG-001 (10 μM) for 24 hours, followed by treatment with palmitic acid (200 μM) or PBS for 8 hours. Finally, the cells were harvested for Western blot analysis. **l**, cells were transfected with indicated constructs for 72 hours and then were collected for western blot analysis. **m**, cells were transfected with indicated constructs for 24 hours and then were collected for western blot analysis. **n**, cells were transfected with indicated constructs for 72 hours followed by treatment with palmitic acid (200 μM) or PBS for 8 hours. Finally, the cells were harvested for Western blot analysis. **o**, Huh7 and Hep3B cells were collected and ChIP-qPCR analysis was performed using IgG or SP5 antibodies. **p**, Huh7 and Hep3B cells were collected for luciferase reporter assays.

Supplementary figure 6

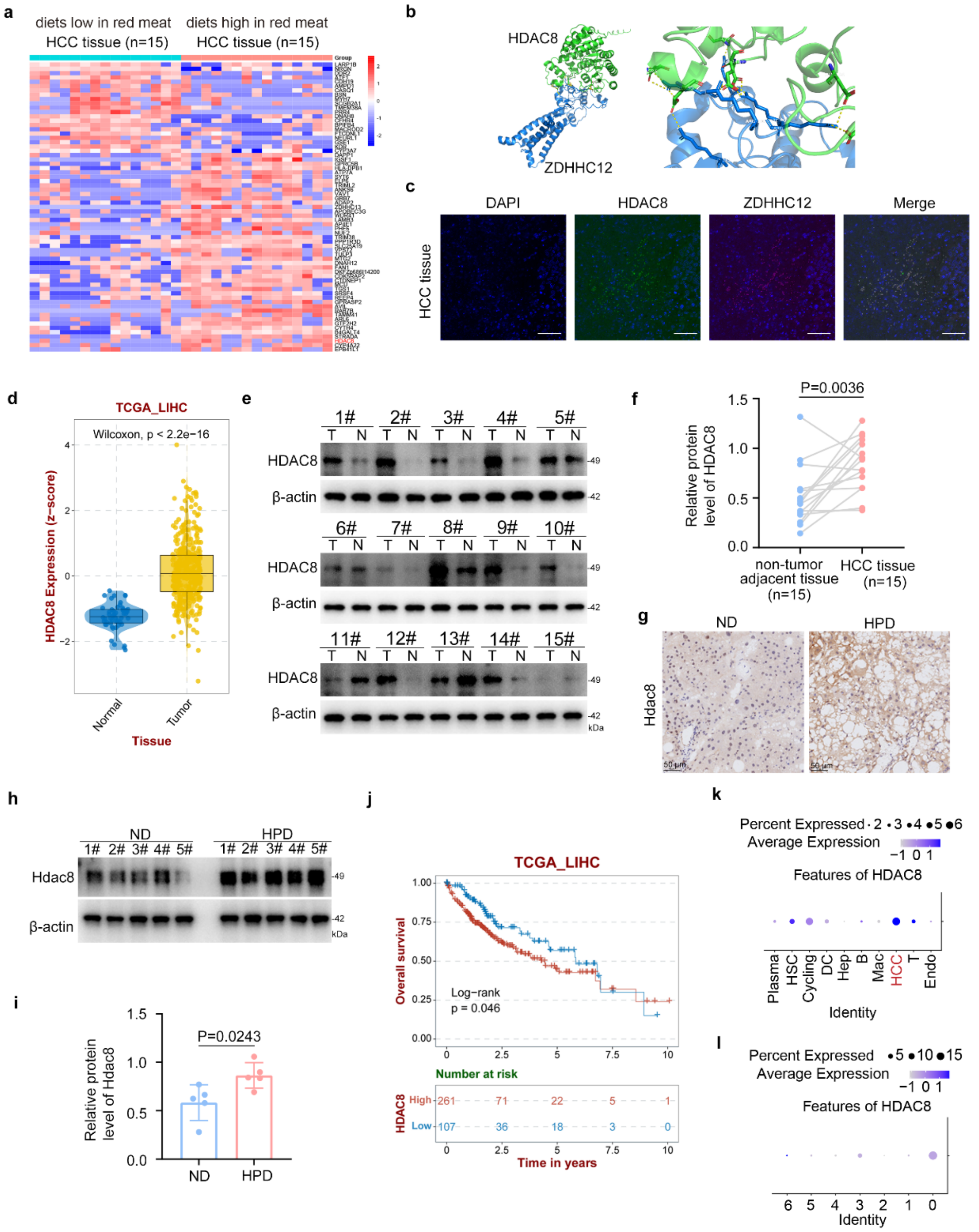

Supplementary figure 6 (related to figure 3). a, HCC tissues from15 HCC patients with high red meat diet and 15 HCC patients with normal diet were collected for 4D-label free quantitative proteomics assay. **b**, molecular docking of the protein structure predicted by alphafold3 indicated the presence of binding between ZDHHC12 and HDAC8. **c**, immunofluorescence co-localization analysis of ZDHHC12 and HDAC8 in HCC tissues. **d**, HDAC8 expression in TCGA-LIHC, which reflects the difference in expression

between normal and hepatocellular carcinoma tissues at the transcriptional level. TCGA data analysis was performed using the BEST tool (https://rookieutopia.hiplot.com.cn/app_direct/BEST/). **e and f**, western blot analysis reflected the HDAC8 protein expression levels in hepatocellular carcinoma tissues and adjacent non-tumor tissues (n=15). **g**, representative IHC images of HDAC8 protein in the livers of normal diet or high fat diet fed mice. **h and i**, western blot analysis reflected the HDAC8 protein content in the livers of normal diet or high palmitic acid diet fed mice. **j**, survival curves of hepatocellular carcinoma patients (TCGA-LIHC) between HDAC8 high and low expression groups. TCGA data analysis was performed using the BEST tool (https://rookieutopia.hiplot.com.cn/app_direct/BEST/). **k**, Expression profile of HDAC8 across diverse cell types. **l,** Expression levels of HDAC8 in all HCC and Hep subpopulation cells across multiple refined subgroups.

Supplementary figure 7

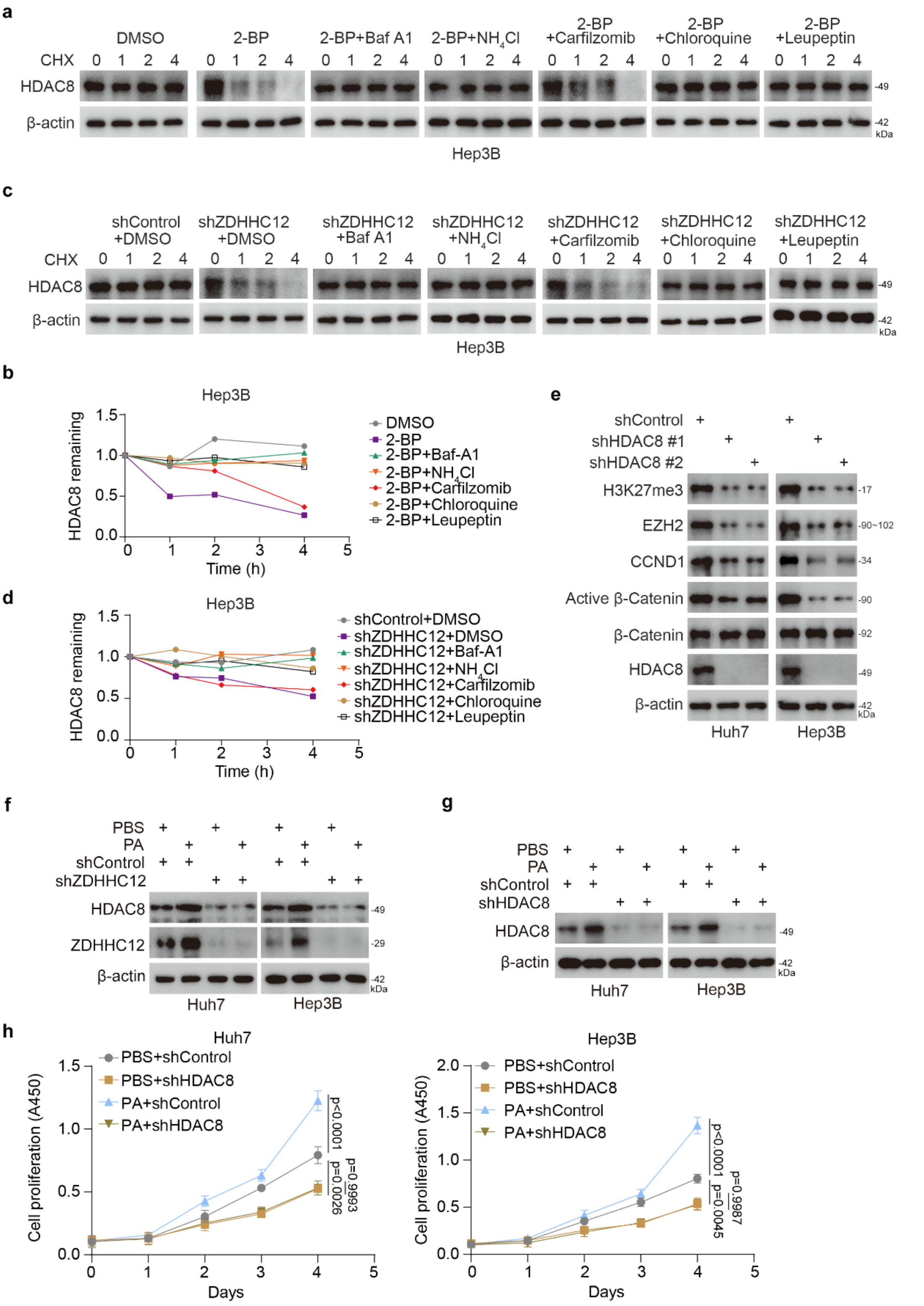

Supplementary figure 7 (related to figure 3). a and b, degradation of HDAC8 in Hep3B cells treated with or without 2-BP was determined by the addition of CHX in the presence of lysosomal inhibitors (Baf A1, NH4Cl, Chloroquine and Leupeptin) and the proteasome inhibitor carfilzomib. The graph on the right shows the changes in the relative residual levels of HDAC8. **c and d,**

degradation of HDAC8 in Huh7 cells treated with indicated constructs was determined by the addition of CHX in the presence of lysosomal inhibitors (Baf A1, NH4Cl, Chloroquine and Leupeptin) and the proteasome inhibitor carfilzomib. The graph on the right shows the changes in the relative residual levels of HDAC8. **e**, cells were transfected with indicated constructs for 72 hours and then were collected for western blot analysis. **f**, cells were transfected with indicated constructs for 72 hours. The cells were treated with PBS or palmitic acid (200 μM) and collected for western blot analysis. **g and h**, cells were transfected with shControl or shHDAC8 plasmids. Puromycin selection was initiated 72 hours post-transfection. After successful selection, stably transfected cells were collected. The cells were treated with PBS or palmitic acid (200 μM) and collected for western blotting analysis (g) and CCK-8 assays (h).

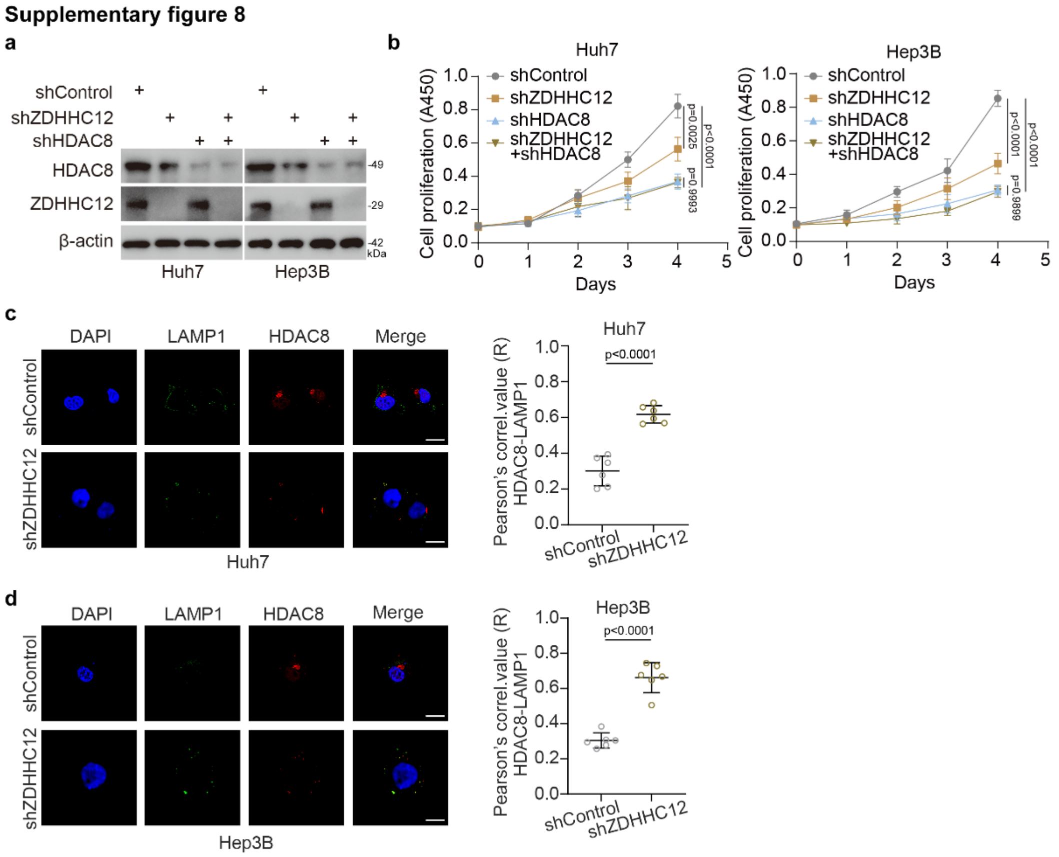

Supplementary figure 8 (related to figure 5). a-d, cells were transfected with indicated plasmids. Puromycin selection was initiated 72 hours post-transfection. After successful selection, stably transfected cells were collected for western blotting (a) and CCK-8 (b) assays. Cells from the shControl and shZDHHC12 groups were collected for immunofluorescence staining and confocal

microscopy imaging. representative images of HDAC8, LAMP1 and DAPI immunofluorescence staining in Huh7 (c) and Hep3B (d) cells with or without ZDHHC12 knockdown. Scale bar: 7.5 μm; n = 6 biologically independent experiments, two-tailed unpaired t test.

Supplementary figure 9

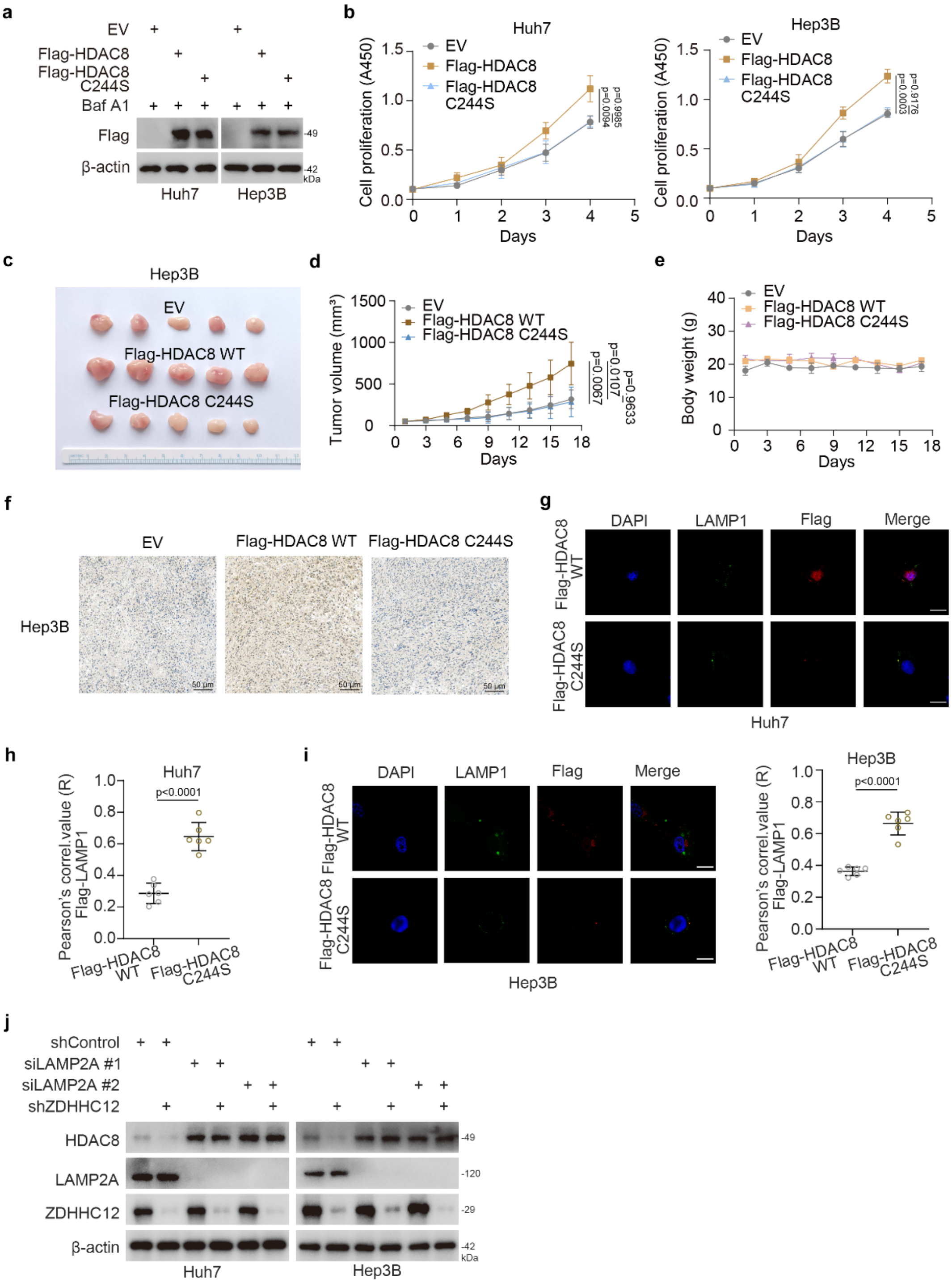

Supplementary figure 9 (related to figure 5). a-i, cells were transfected with indicated plasmids. Puromycin selection was initiated 24 hours post-transfection. Following successful selection, a portion of the stably transfected cells were treated with Baf A1 for 6 hours and then harvested for Western blot analysis (a), while the remaining stably transfected cells were reserved for subsequent experiments. The stably transfected Huh7 and Hep3B cells were utilized for CCK-8 assays (b). Stably transfected Hep3B cells were subcutaneously injected into the flanks of nude mice, and tumors were excised at appropriate time points for photographic documentation (c). Collected measurement data were used to plot growth curves (d) and body weight change curves (e). The tumor tissues were subjected to IHC staining (f). Stably transfected Huh7 and Hep3B cells were employed for immunofluorescence staining and confocal microscopy imaging. Representative images of HDAC8, LAMP1 and DAPI immunofluorescence staining in Huh7 (g and h) and Hep3B (i) cells with or without ZDHHC12 knockdown. Scale bar: 7.5 μm; n = 6 biologically independent experiments, two-tailed unpaired t test. **j**, cells were transfected with indicated constructs for 72 hours and then were collected for western blot analysis.

Supplementary figure 10

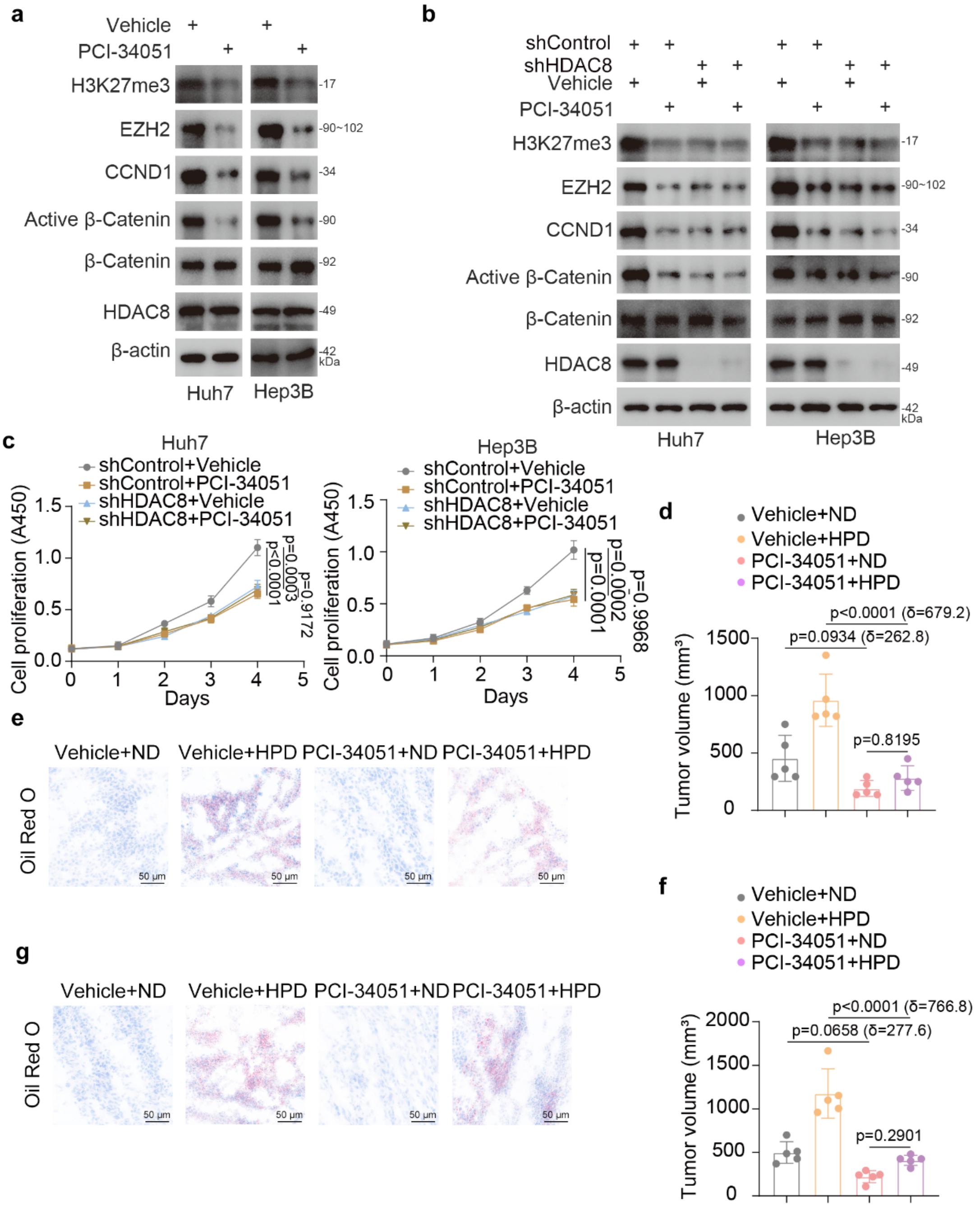

Supplementary figure 10 (related to figure 6). a, Huh7 and Hep3B cells were treated with PCI-34051 (10 μM) for 24 hours, followed by cell collection and Western blotting analysis. **b**, Huh7 and Hep3B cells were transfected with indicated constructs for 72 hours followed by being treated with PCI-34051 (10 μM) for 24 hours and collected for western blotting analysis. **c**, cells were transfected with indicated plasmids. Puromycin selection was initiated 72 hours post-transfection. After successful selection, stably transfected cells were collected and treated with PCI-34051 (10 μM) for

24 hours. Then the cells were collected for western blotting assays. **d and e**, tumor tissues from hepatocellular carcinoma patients on a high saturated fatty acid diet were used to construct a PDX model. The tumor-bearing nude mice were treated with vehicle or PCI-34051 (10 mg/kg/day, every three days). Tumor volume statistics (d). Representative Oil Red O staining images of tumor tissues (e). δ is the meaning of the absolute value of the difference between the two groups. **f and g**, Representative Oil Red O staining images of Huh7 cell-derived xenograft (CDX) models fed with either a high-palmitic acid diet or normal diet, with or without PCI-34051 treatment. Tumor volume statistics (f). Representative Oil Red O staining images of tumor tissues (g). δ is the meaning of the absolute value of the difference between the two groups.

Supplementary figure 11

a

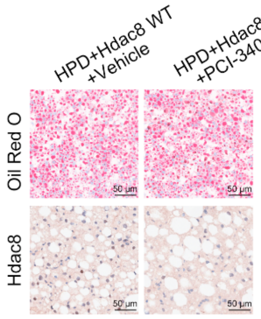

b

Strain	primer	Primer sequence（5' → 3'）			Product (bp)
Hdac8-flox	YDS0944-1A_ HDAC8	CTGCTCATACACACATGGAACTAA	Forward	A	WT=248 bp
	YDS0944-2A_ HDAC8	TATTAAGGTTAGGGAGGTAGCTCAG	Reverse	A	Mutant=315 bp
Alb-Cre	YDS0223-1A_Alb	TGCAAACATCACATGCACAC	Wild type Forward	A	
	YDS0223-2A_Alb	TTGGCCCCTTACCATAACTG	Common Reverse	A	WT = 351 bp
	YDS0223-3B_Alb	TTGGCCCCTTACCATAACTG	Common Reverse	B	Mutant = 390 bp
	YDS0223-4B_Alb	GAAGCAGAAGCTTAGGAAGATGG	Mutant Forward	B	

c

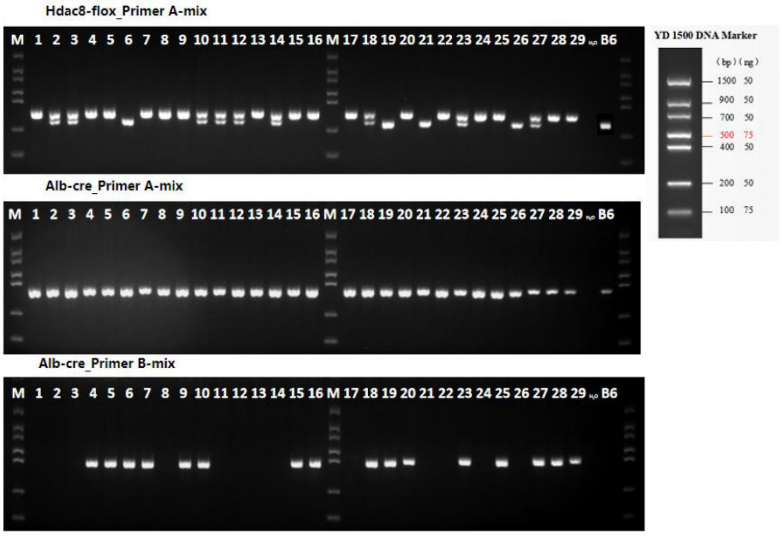

d

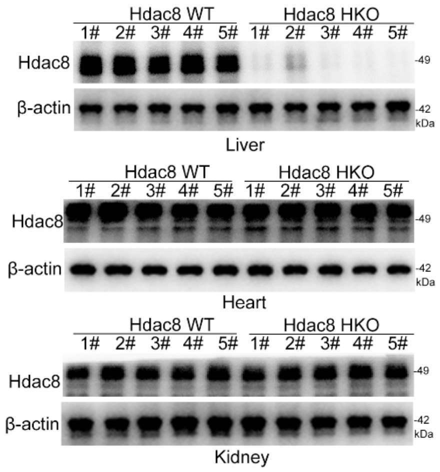

Supplementary figure 11 (related to figure 6). a, liver cancer was induced by tail vein injection of pT3-myr-Akt-HA and pCaggs-NRasV12 plasmids in Hdac8 WT mice fed with high palmitic acids diet (HPD) for 12 weeks. This was followed by treatment of vehicle or PCI-34051 (10 mg/kg/d,

i.p., per day) for 10 days. Representative Oil Red O staining and immunohistochemical (IHC) images of tumor tissues. **b**, Cre and LoxP DNA sequences used in knockout Hdac8 in mouse liver. **c**, The tail end of the mouse was cut about 0.5 cm, and the tissue was lysed to extract DNA, which was amplified by PCR and then electrophoresed to identify the genetically engineered mice. **d**, western blot analysis of Hdac8 protein expression in various organs of Hdac8 wild-type and liver Hdac8 knockout mice.

Supplementary figure 12

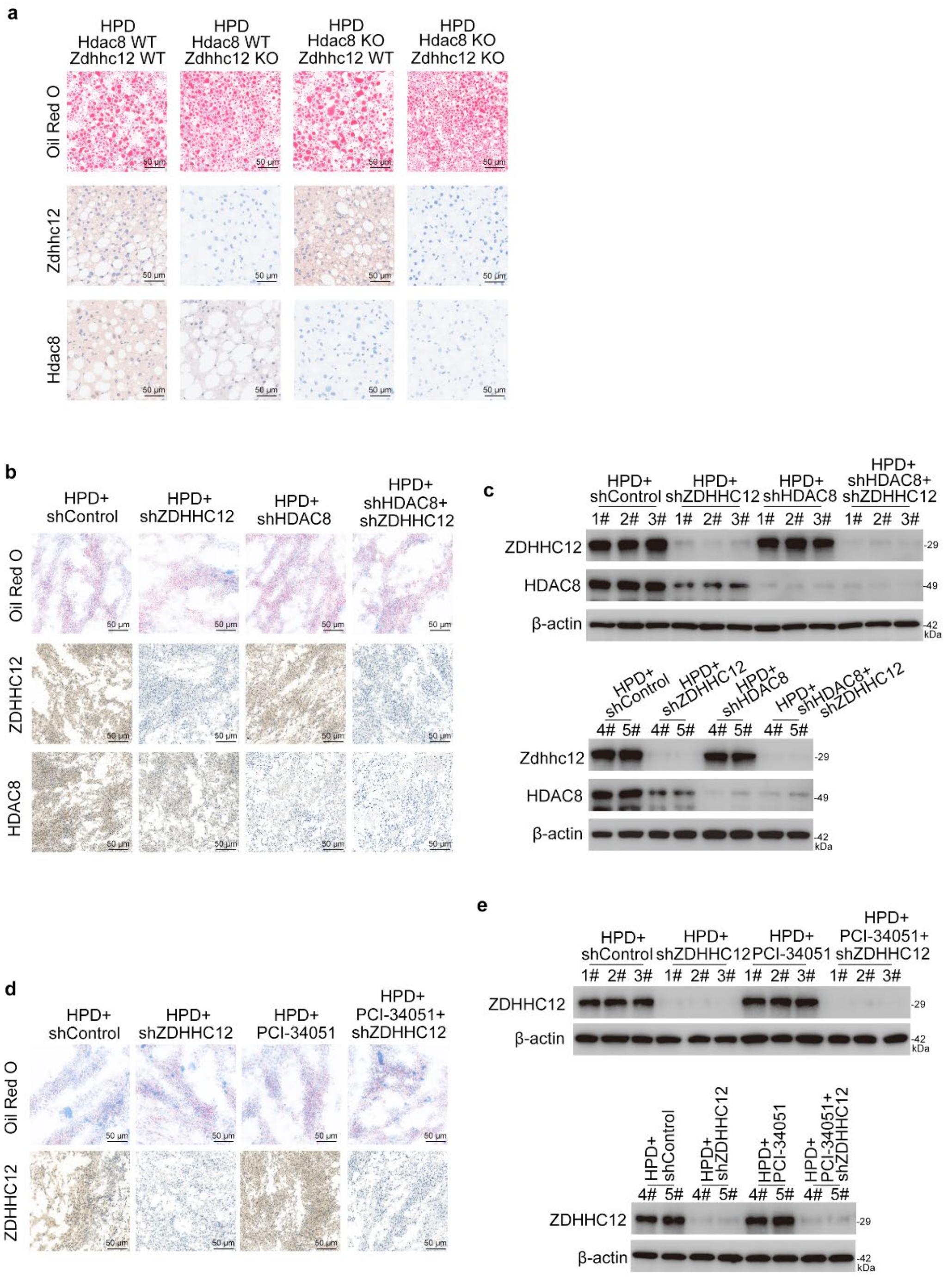

Supplementary figure 12 (related to figure 6). a, liver cancer was induced in Hdac8 WT/KO or Zdhhc12 WT/KO mice fed with high palmitic acids diet (HPD) for 12 weeks. Mouse livers were then collected and subjected to Oil Red O staining and immunohistochemical staining. **b and c,** Hep3B cells were transfected with indicated constructs. Puromycin selection was initiated 72 hours post-transfection. After successful selection, stably transfected cells were collected and subcutaneously injected into the dorsal flank of nude mice had fed with high palmitic acids diet or

normal diet for 12 weeks. After 19 days, the tumors were collected for IHC (b) and western blotting (c) analysis. **d and e,** Hep3B cells were transfected with indicated constructs. Puromycin selection was initiated 72 hours post-transfection. After successful selection, stably transfected cells were collected and subcutaneously injected into the dorsal flank of nude mice that had been fed a High Palmitic Acid Diet for 12 weeks. When tumors reached 50 mm^3, mice were injected with PCI-34051 (10 mg/kg/d, i.p.) every three days. After 19 days, the tumors were collected for IHC (d) and western blotting (e) analysis.

Table S1 Patient Basic Information

Patient ID	Type	Gender	Age	HBsAg	AFP (ng/ml)	Cirrhosis	Tumor size(cm)	Tumor Stage	Histology Type	Histologic grade	TNM Groups	MVI	Weight(Kg)	Sampling site	Smoking status	Drinking status	Total daily calorie intake(kcal)	Fiber consumption every day(g)	Exercise frequency (times/week)	Dietary pattern
1	HCC	Male	51	(-)	11.54	no	2.5x2.3x1.8	Ia	Hepatocellular carcinoma	G2	T1bN0M0	0	78	Tumor	Non-smoker	Non-drinker	1872	18	3	normal diet
2	HCC	Male	54	(-)	5.31	yes	2.9x1.5x1	Ib	Hepatocellular carcinoma	G2	T2N0M0	0	84	Tumor	Non-smoker	Non-drinker	2150	26	1	normal diet
3	HCC	Male	60	(-)	>800	yes	5x3.5x3.4	Ib	Hepatocellular carcinoma	G3	T1bN0M0	0	77	Tumor	Non-smoker	Non-drinker	2435	32	3	normal diet
4	HCC	Female	47	(-)	26.51	no	3x3x3	Ia	Hepatocellular carcinoma	G2	T1bN0M0	0	68	Tumor	Smoker	Drinker	1689	17	6	normal diet
5	HCC	Male	73	(-)	89.97	no	9x6x6	IV	Hepatocellular carcinoma	G3	T2N0M0	0	79	Tumor	Smoker	Non-drinker	2780	29	1	normal diet
6	HCC	Male	56	(-)	5.02	yes	3.5x2.5x2.5	Ia	Hepatocellular carcinoma	G1	T1bN0M0	0	86	Tumor	Non-smoker	Non-drinker	1945	22	7	normal diet
7	HCC	Female	66	(-)	2.11	no	6x5x4.8	Ib	Hepatocellular carcinoma	G1	T1bN0M0	0	64	Tumor	Smoker	Drinker	2260	35	2	normal diet
8	HCC	Female	50	(-)	759	yes	5x5x3.7	IIIa	Hepatocellular carcinoma	G4	T2N0M0	1	54	Tumor	Non-smoker	Non-drinker	1540	20	2	normal diet
9	HCC	Male	48	(-)	193.51	no	2x1.2x1	Ia	Hepatocellular carcinoma	G1	T1aN0M0	0	70	Tumor	Smoker	Drinker	2615	27	1	normal diet
10	HCC	Female	60	(-)	214	yes	4.5x4x3	IIIa	Hepatocellular carcinoma	G2	T2N0M0	1	63	Tumor	Non-smoker	Non-drinker	2030	24	7	normal diet
11	HCC	Male	57	(-)	3.36	yes	3.5x3.2x2.2	Ia	Hepatocellular carcinoma	G2	T1bN0M0	0	75	Tumor	Non-smoker	Drinker	2350	30	6	normal diet
12	HCC	Female	60	(-)	2.34	no	3x2.8x2.5	IIIa	Hepatocellular carcinoma	G2	T2N0M0	1	72	Tumor	Smoker	Drinker	1720	17	5	normal diet
13	HCC	Male	73	(-)	4.33	yes	6x6.5x5	Ib	Hepatocellular carcinoma	G1	T1bN0M0	0	60	Tumor	Smoker	Drinker	2880	39	0	normal diet
14	HCC	Female	74	(-)	22.84	no	4.5x4.5x4	Ia	Hepatocellular carcinoma	G2	T1bN0M0	0	57	Tumor	Smoker	Drinker	1980	21	3	normal diet
15	HCC	Male	52	(-)	50.89	no	9.6x8.5x7	IIIa	Hepatocellular carcinoma	G3	T2N0M0	1	67	Tumor	Smoker	Drinker	2210	27	6	normal diet
16	HCC	Male	65	(-)	19	yes	12x10x8.5	IIIa	Hepatocellular carcinoma	G3	T2N0M0	1	80	Tumor	Smoker	Non-drinker	1620	16	6	high-red-meat diet
17	HCC	Male	47	(-)	>800	yes	3.7x3.2x3.5	Ia	Hepatocellular carcinoma	G2	T1bN0M0	0	84	Tumor	Non-smoker	Drinker	2520	34	0	high-red-meat diet
18	HCC	Female	49	(-)	2.82	yes	1.8x1x1	Ia	Hepatocellular carcinoma	G1	T1aN0M0	0	51	Tumor	Smoker	Non-drinker	2070	23	3	high-red-meat diet
19	HCC	Male	51	(-)	118	yes	10x7x9	Ib	Hepatocellular carcinoma	G2	T1bN0M0	0	59	Tumor	Non-smoker	Drinker	2300	29	2	high-red-meat diet
20	HCC	Female	58	(-)	105	yes	8x6x4	Ib	Hepatocellular carcinoma	G2	T1bN0M0	0	57	Tumor	Smoker	Drinker	1810	21	2	high-red-meat diet
21	HCC	Female	56	(-)	>800	yes	4.5x3x3	IIIa	Hepatocellular carcinoma	G3	T2N0M0	1	88	Tumor	Smoker	Drinker	2650	36	5	high-red-meat diet
22	HCC	Male	49	(-)	41.24	no	6x5x4	Ib	Hepatocellular carcinoma	G2	T1bN0M0	0	68	Tumor	Non-smoker	Drinker	1920	19	6	high-red-meat diet
23	HCC	Female	58	(-)	0.97	no	0.4x0.3x0.1	Ia	Hepatocellular carcinoma	G2	T1aN0M0	0	81	Tumor	Non-smoker	Drinker	2410	32	6	high-red-meat diet
24	HCC	Male	51	(-)	>800	yes	5x4.5x4	IIIa	Hepatocellular carcinoma	G3	T2N0M0	1	79	Tumor	Smoker	Drinker	1750	16	1	high-red-meat diet
25	HCC	Male	39	(-)	199.34	yes	4x3x2.5	IIIa	Hepatocellular carcinoma	G2	T2N0M0	1	56	Tumor	Smoker	Non-drinker	2710	40	6	high-red-meat diet
26	HCC	Male	52	(-)	>800	no	15x11x10	IIIa	Hepatocellular carcinoma	G2	T2N0M0	2	61	Tumor	Non-smoker	Non-drinker	2100	23	3	high-red-meat diet
27	HCC	Male	55	(-)	66.75	yes	4.4x4x3	Ia	Hepatocellular carcinoma	G1	T1bN0M0	0	54	Tumor	Smoker	Non-drinker	2480	35	4	high-red-meat diet
28	HCC	Male	54	(-)	355	yes	11x9x7	IIIa	Hepatocellular carcinoma	G3	T2N0M0	2	70	Tumor	Non-smoker	Drinker	1580	15	4	high-red-meat diet
29	HCC	Male	69	(-)	1.98	yes	1.5x1.5x1	Ia	Hepatocellular carcinoma	G1	T1aN0M0	0	68	Tumor	Non-smoker	Non-drinker	2850	42	6	high-red-meat diet
30	HCC	Male	61	(-)	32.4	no	3.5x3x2.5	IIIa	Hepatocellular carcinoma	G2	T1bN0M0	1	69	Tumor	Non-smoker	Non-drinker	2250	28	2	high-red-meat diet

Abbreviations:HCC=hepatocellular carcinoma; AFP=alpha-fetoprotein; MVI=microvascular invasion.

Table S 2 Descriptive Analysis

Variable	Description (n=30)	The amount (proportion) of missing data
Gender		0 (0.000)
Male	20 (66.667%)	
Female	10 (33.333%)	
Age	56.500±8.492<br>55.500 (51.000, 60.000)	0 (0.000)
Weight(Kg)	69.300±10.665<br>68.500 (60.250, 78.750)	0 (0.000)
Smoking status		0 (0.000)
Non-smoker	15 (50.000%)	
Smoker	15 (50.000%)	
Drinking status		0 (0.000)
Non-drinker	14 (46.667%)	
Drinker	16 (53.333%)	
Total daily calorie intake	2182.533±395.057<br>2180.000 (1884.000, 2468.750)	0 (0.000)
Dietary consumption every day	26.360±7.821<br>26.150 (19.750, 31.950)	0 (0.000)
Exercise frequency (times/week)	3.633±2.205<br>3.000 (2.000, 6.000)	0 (0.000)
dietary pattern		0 (0.000)
normal diet	15 (50.000%)	
high-red-meat diet	15 (50.000%)	

Table S 3 Baseline Statistical Table

Variable	Total(n=30)	dietary pattern		Statistical magnitude	*P-value*	SMD
		normal diet (n=15)	high-red-meat diet (n=15)			
Gender, n (%)				-[1]	0.700	0.286
Male	20 (66.667)	9 (60.000)	11 (73.333)			
Female	10 (33.333)	6 (40.000)	4 (26.667)			
Age, Mean±SD	56.500±8.492	58.733±9.122	54.267±7.450	t=1.469[2]	0.153	0.555
Weight(Kg), Mean±SD	69.300±10.665	70.267±9.625	68.333±11.872	t=0.490[2]	0.628	0.185
Smoking status, n (%)				-[1]	1.000	0.134
Non-smoker	15 (50.000)	7 (46.667)	8 (53.333)			
Smoker	15 (50.000)	8 (53.333)	7 (46.667)			
Drinking status, n (%)				-[1]	1.000	0.000
Non-drinker	14 (46.667)	7 (46.667)	7 (46.667)			
Drinker	16 (53.333)	8 (53.333)	8 (53.333)			
Total daily calorie intake, Mean±SD	2182.533±395.057	2163.733±397.515	2201.333±405.601	t=-0.256[2]	0.800	0.097
Dietary consumption every day, M (Q_1, Q_3)	26.150 (19.750, 31.950)	25.500 (20.250, 29.450)	27.900 (19.700, 34.150)	W=103.000[3]	0.713	0.211
Exercise frequency (times/week), M (Q_1, Q_3)	3.000 (2.000, 6.000)	3.000 (1.500, 6.000)	4.000 (2.000, 6.000)	Z=-0.211[3]	0.833	0.092

Notes: 1. Fisher's exact probability method; 2. Independent sample t-test; 3. Continuity-corrected Mann Whitney U test.

Table S4. The siRNA, shRNA and sgRNA sequences.

shZDHHC12 #1	5'- GATCGGTCAGTGGTTGCGGTCCATTCTCGAG AATGGACCGCAACCACTGACCTTTTTG -3'
shZDHHC12 #2	5'- GATCAGGAGGAGCTCAAAGAGGATTCTCGAG AATCCTCTTTGAGCTCCTCCTTTTTG -3'
shHDAC8 #1	5'- GATCGGAAGTATACCAAGCCTTTAACTCGAGTTAAAGGCTTGGTATACTTCC TTTTTG -3'
shHDAC8 #2	5'- GATCGCAAGTGTCTTAAGTACATCCCTCGAGGGATGTACTTAAGACACTTGC TTTTTG -3'
shSMARCA4 #1	5'- GATCGGCATAGGCCTTAGCAGTAACCTCGAGGTTACTGCTAAGGCCTATGC CTTTTTG -3'
shSMARCA4 #1	5'- GATCGTACCGAGCCTCGGGTAAATTCTCGAGAATTTACCCGAGGCTCGGTA CTTTTTG -3'
siHSC70 #1	5'- CGUCUGAUUGGACGCAGAUUUTT -3'
siHSC70 #2	5'- CCAAGACUUCUUCAAUGGAAATT -3'
shBRCA1 #1	5'- GATCGAGTATGCAAACAGCTATAATCTCGAG ATTATAGCTGTTTGCATACTCTTTTTG -3'
shBRCA1 #2	5'- GATCTTGCAACCTGAGGTCTATAAACTCGAGTTTATAGACCTCAGGTTGCA ATTTTTG -3'
shSP5 #1	5'- GATCCGAAGCACGTCAAGACTCACTCGAGTGAGTCTTGACGTGCTTCGTT TTTG -3'
shSP5 #2	5'- GATCACGAGTTCTCGCCGGTCAACTCGAGTTGACCGGCGAGAACTCGTTT

	TTTG -3'
siLAMP2A #1	5'- GAAGUGAACAUCAGCAUGUAUTT -3'
siLAMP2A #2	5'- GCCAUCAGAAUUCCAUUGAAUTT -3'
sgZDHHC12	5′- AGTATCTGCAGCGCCGAAGA -3′
sgHDAC8	5'- CGGAGGAACCGGCGGACAGT -3'

Table S5. The primer sequences for RT-qPCR.

**Species**	**Gene**	**Forward (5'-3')**	**Reverse (5'-3')**
Human	β-actin	GCCTCGCCTTTGCCGAT	AGGTAGTCAGTCAGGTCCCG
Human	HDAC8	GAGGAGCAGGAACTGGAACC	AGATGCTTCATCTCTCATCTGCT
Human	ZDHHC12	ACCCTGGCTACGTGAATGTG	TGCAGCACCAGGCAGTATC

Table S6. The primer sequences for ChIP-qPCR.

Gene (Human)	Forward primer (5′ - 3′)	Reverse primer (5′ - 3′)
ZDHHC12 (SMARCA4)	CTGGTGGATCTAGGCGTTGG	CTGTTGGCTTGAGGGTCCAT
SMARCA4 (SP5)	TGCAGAAGGCGAAGCGTG	TGGCCCGGAGGAAGCA
