# Supplementary material for: ZDHHC12 Palmitoylates HDAC8 to Promote the Progression of Hepatocellular Carcinoma Associated with a Diet High in Saturated Fatty Acids
Source: Adv Sci (Weinh). 2025 Aug 11;12(40):e05702. doi: 10.1002/advs.202505702 (PMC12561377; doi:10.1002/advs.202505702)

**Figure 2**

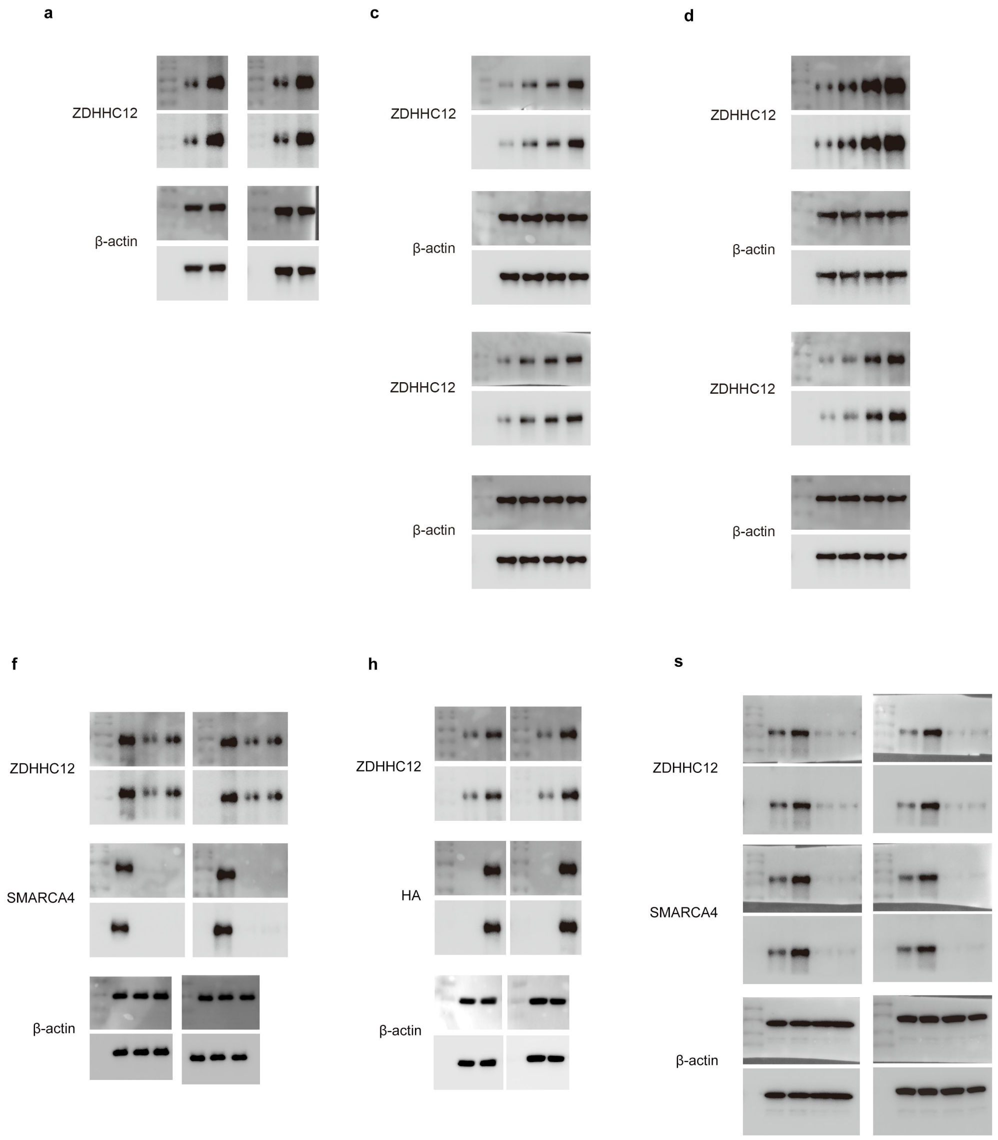
a
ZDHHC12
β-actin
c
ZDHHC12
β-actin
ZDHHC12
β-actin
d
ZDHHC12
β-actin
ZDHHC12
β-actin
f
ZDHHC12
SMARCA4
β-actin
h
ZDHHC12
HA
β-actin
s
ZDHHC12
SMARCA4
β-actin

**Figure 3**

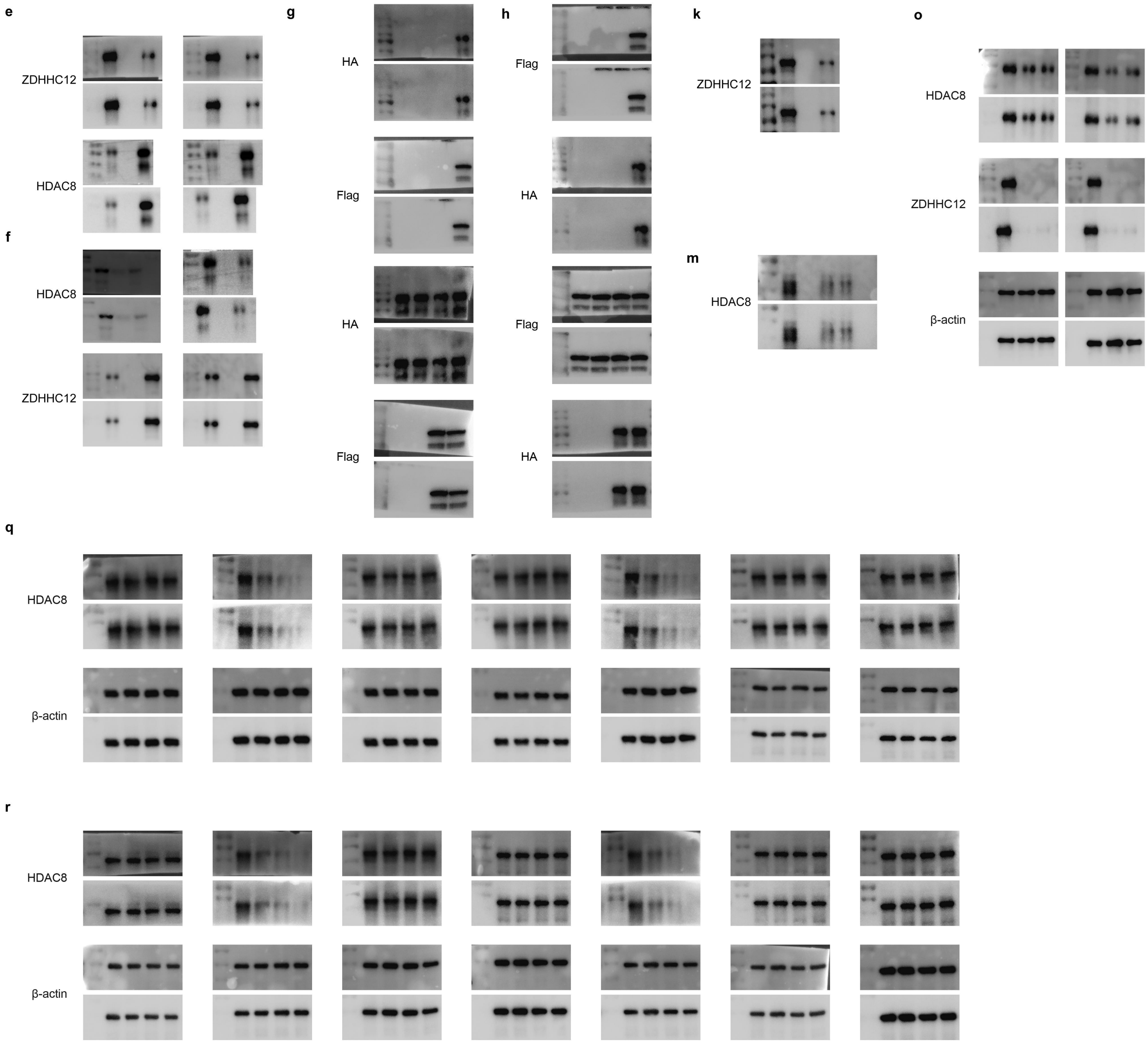

e
ZDHHC12
HDAC8
f
HDAC8
ZDHHC12
g
HA
Flag
HA
Flag
h
Flag
HA
Flag
HA
k
ZDHHC12
m
HDAC8
o
HDAC8
ZDHHC12
β-actin
q
HDAC8
β-actin
r
HDAC8
β-actin

Figure 4

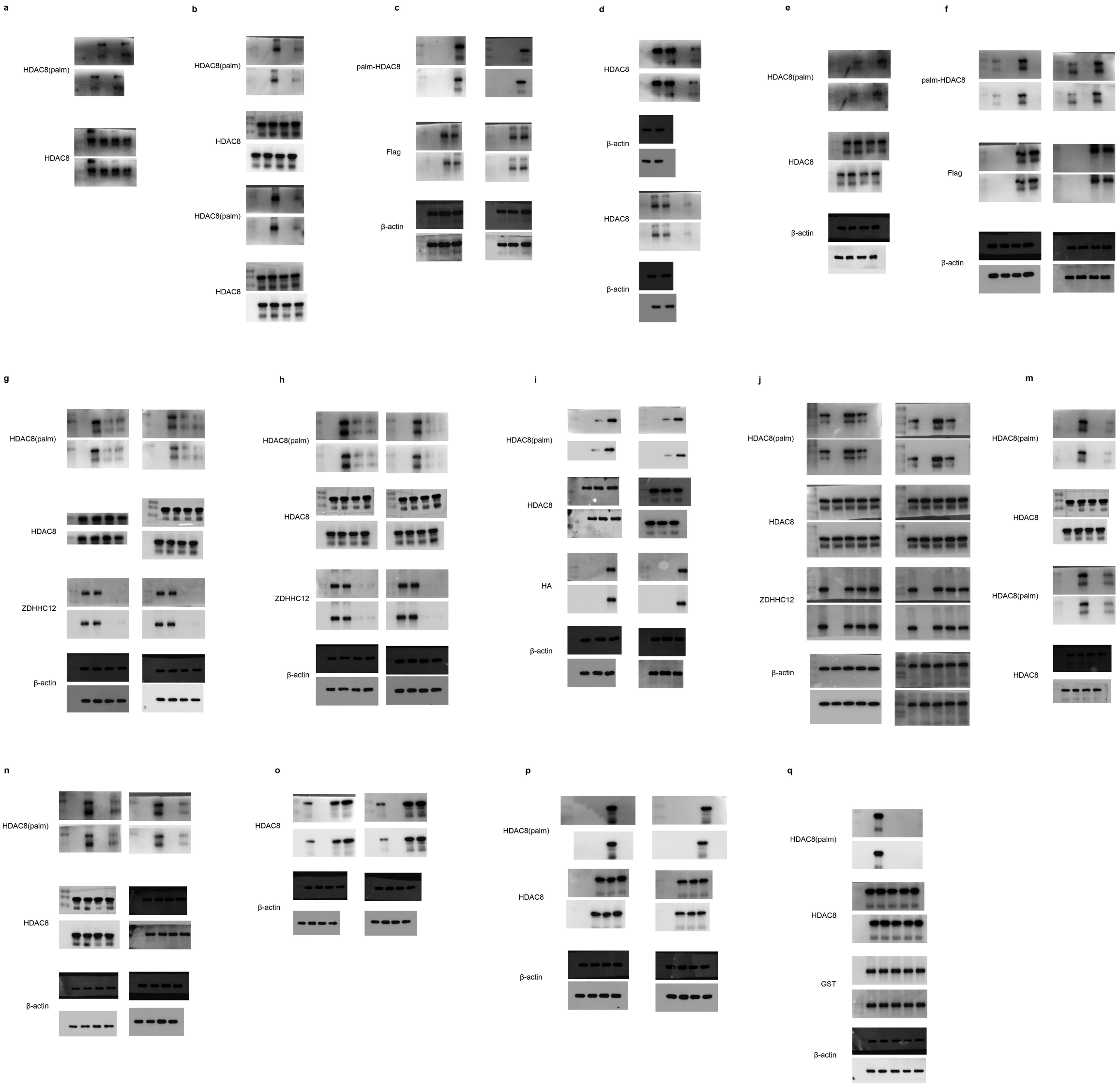
a
HDAC8(palm)
HDAC8
b
HDAC8(palm)
HDAC8
HDAC8(palm)
HDAC8
c
palm-HDAC8
Flag
β-actin
d
HDAC8
β-actin
HDAC8
β-actin
e
HDAC8(palm)
HDAC8
β-actin
f
palm-HDAC8
Flag
β-actin
g
HDAC8(palm)
HDAC8
ZDHHC12
β-actin
h
HDAC8(palm)
HDAC8
ZDHHC12
β-actin
i
HDAC8(palm)
HDAC8
HA
β-actin
j
HDAC8(palm)
HDAC8
ZDHHC12
β-actin
m
HDAC8(palm)
HDAC8
HDAC8(palm)
HDAC8
n
HDAC8(palm)
HDAC8
β-actin
o
HDAC8
β-actin
p
HDAC8(palm)
HDAC8
β-actin
q
HDAC8(palm)
HDAC8
GST
β-actin

**Figure 5**

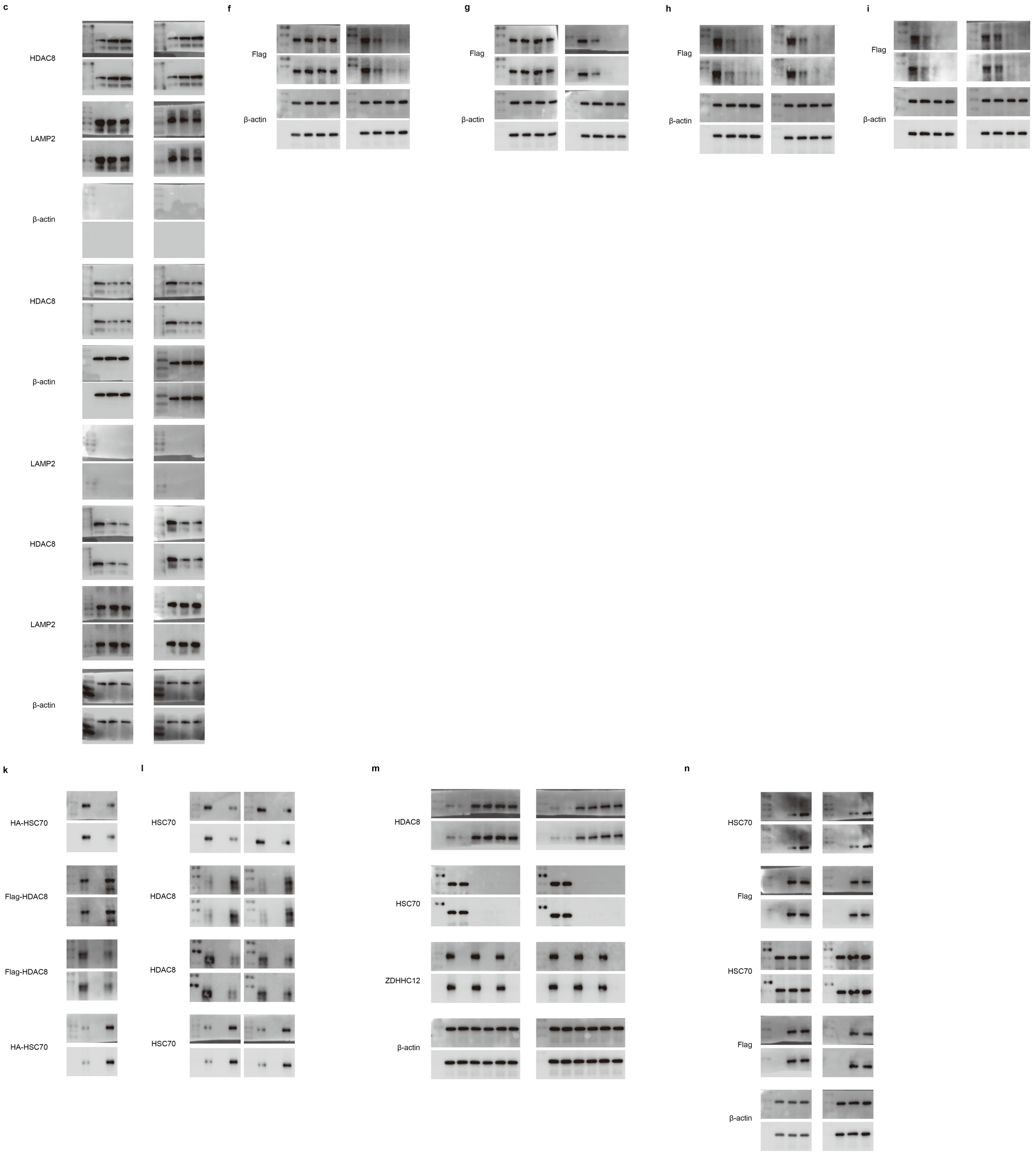
c
HDAC8
LAMP2
β-actin
HDAC8
β-actin
LAMP2
HDAC8
LAMP2
β-actin
f
Flag
β-actin
g
Flag
β-actin
h
Flag
β-actin
i
Flag
β-actin
k
HA-HSC70
Flag-HDAC8
Flag-HDAC8
HA-HSC70
l
HSC70
HDAC8
HDAC8
HSC70
m
HDAC8
HSC70
ZDHHC12
β-actin
n
HSC70
Flag
HSC70
Flag
β-actin

**Supplementary figure 1**

**a**

Zdhhc12

β-actin

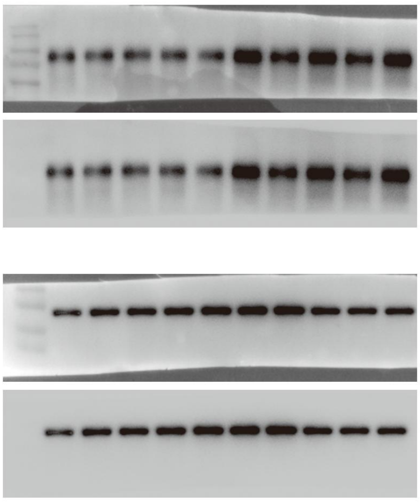

**g**

ZDHHC12

β-actin

ZDHHC12

β-actin

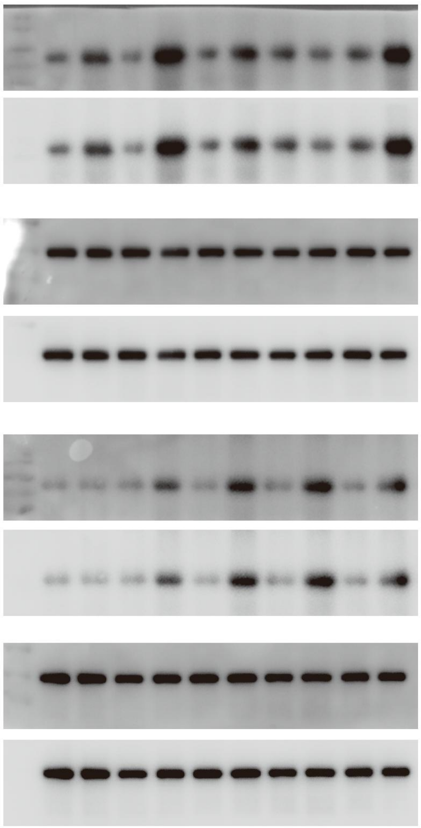

**Supplementary figure 3**

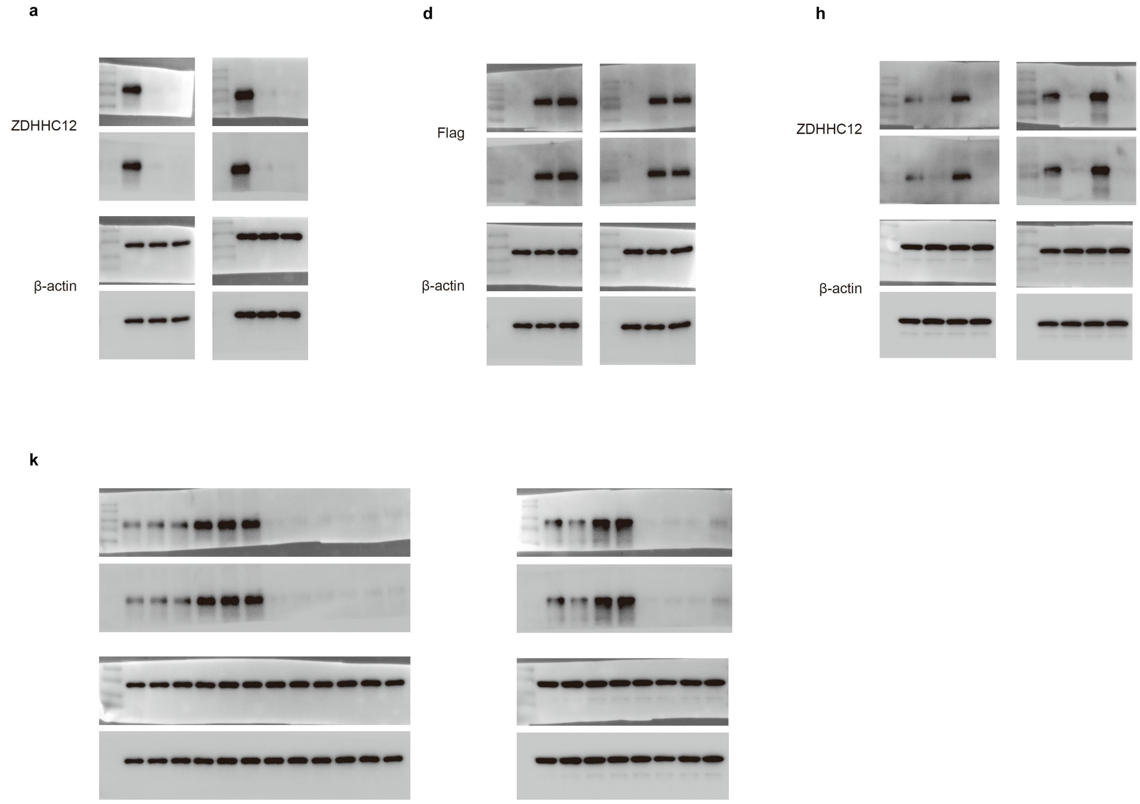

**Supplementary figure 4**

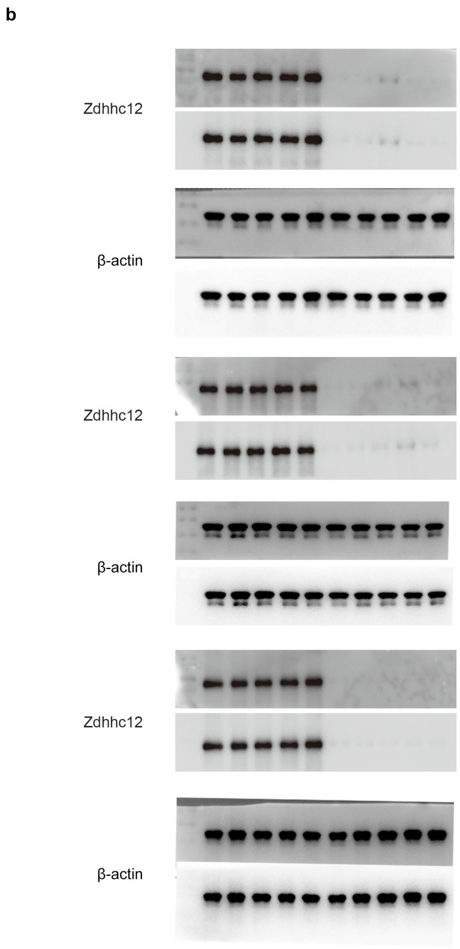


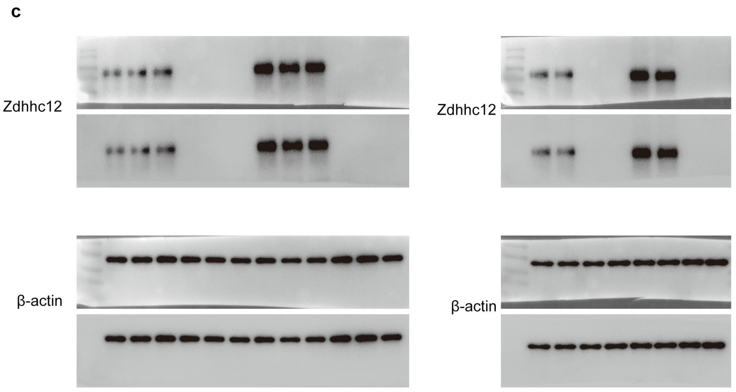

**Supplementary figure 5**

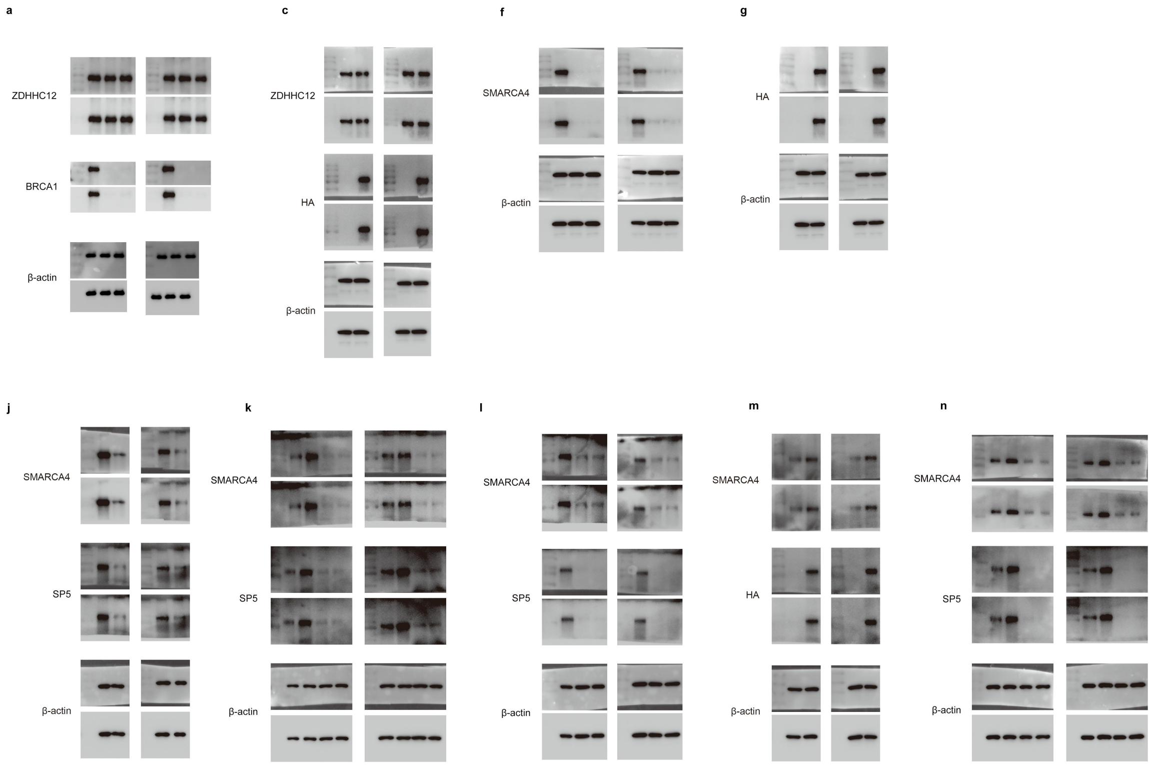

**Supplementary figure 6**

e

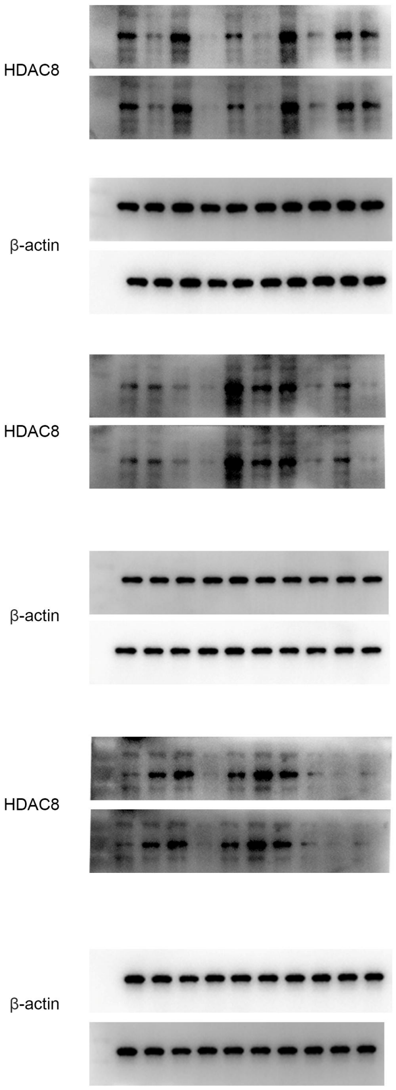


h

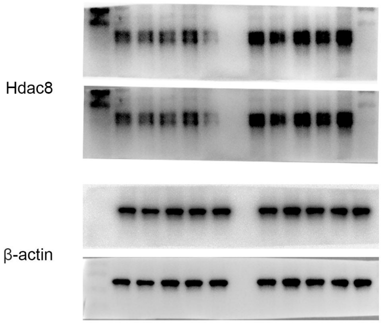

**Supplementary figure 7**

**a**

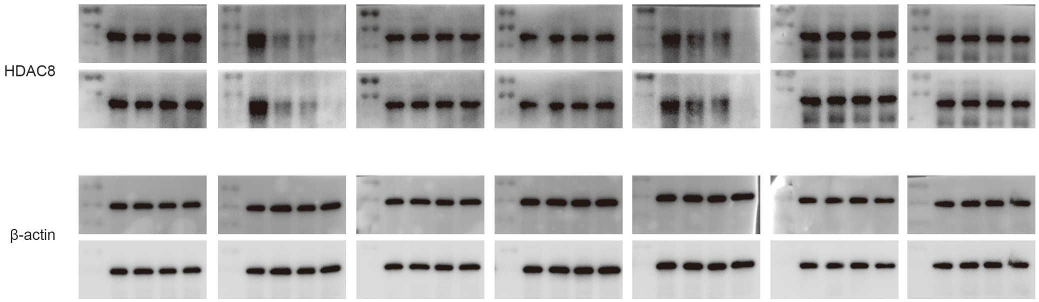


**c**

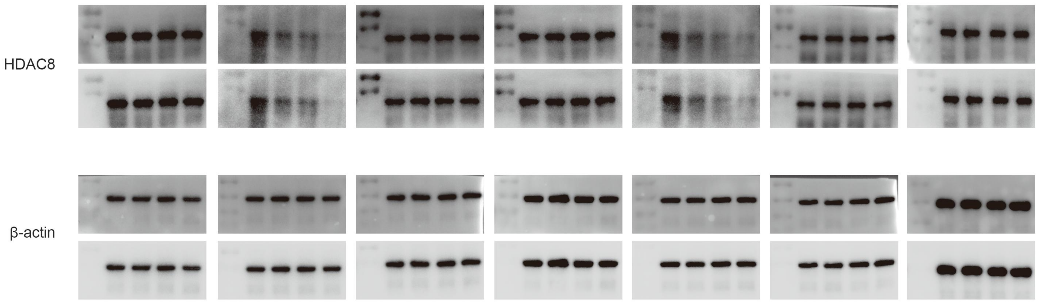


**e**

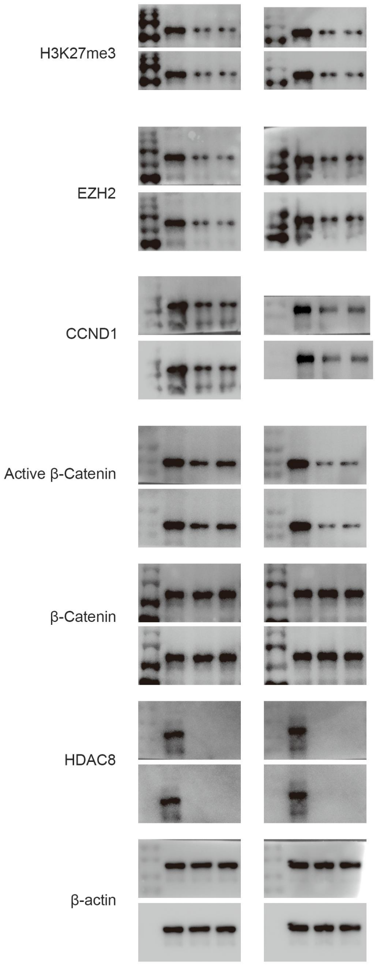


**f**

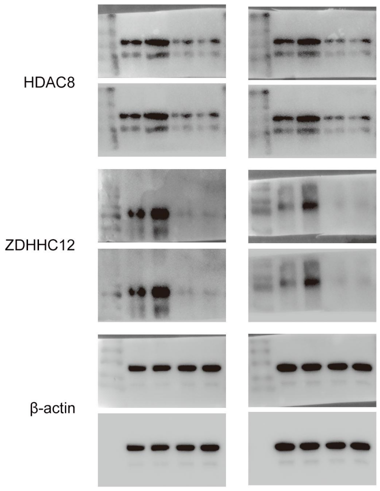


**g**

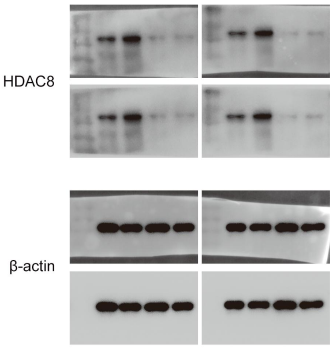

## Supplementary figure 8

**a**

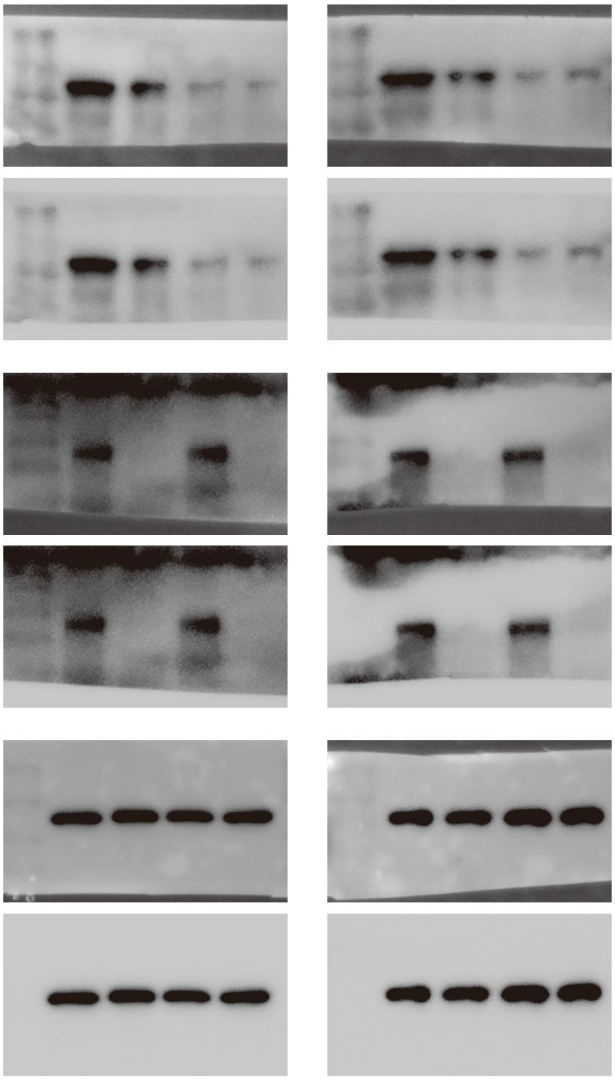

**Supplementary figure 9**

a

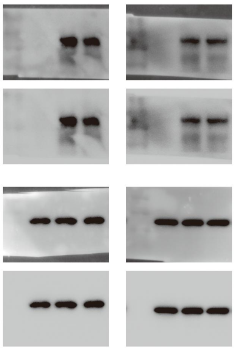

j

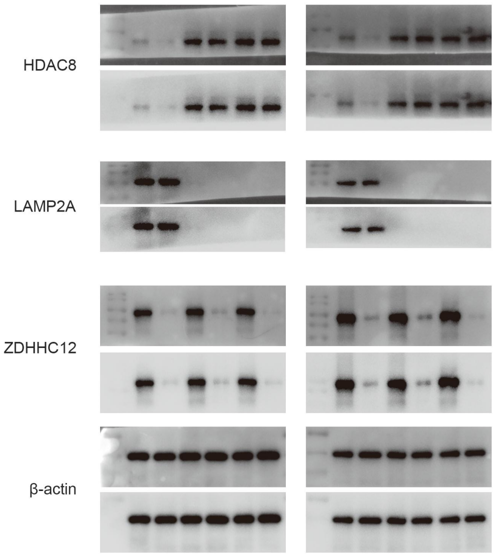

**Supplementary figure 10**

a

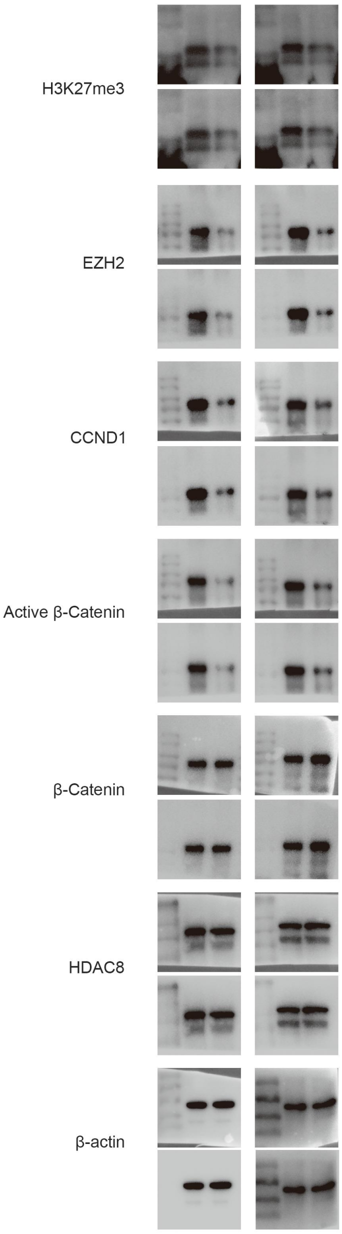


b

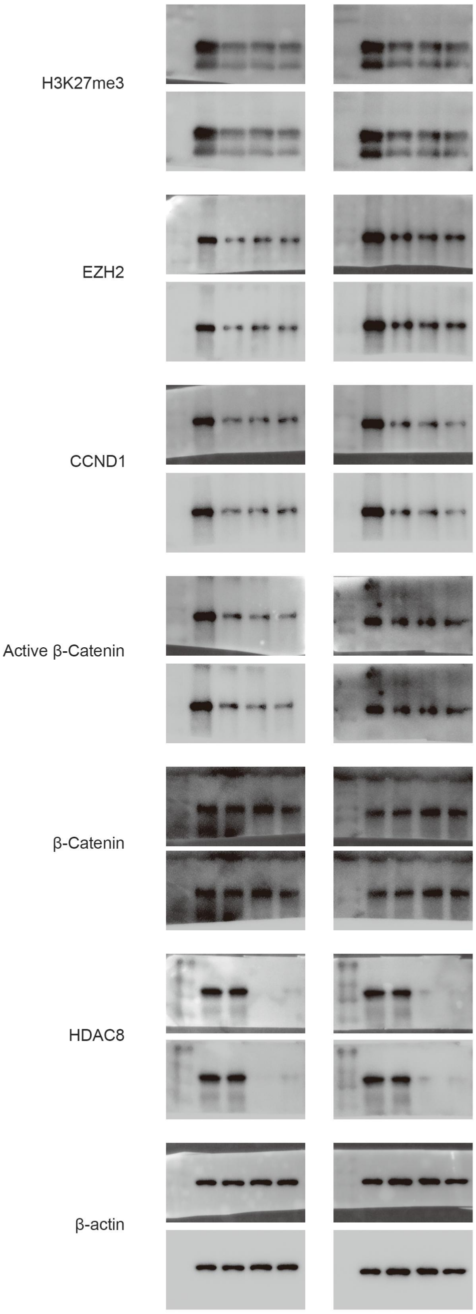

**Supplementary figure 11**

**d**

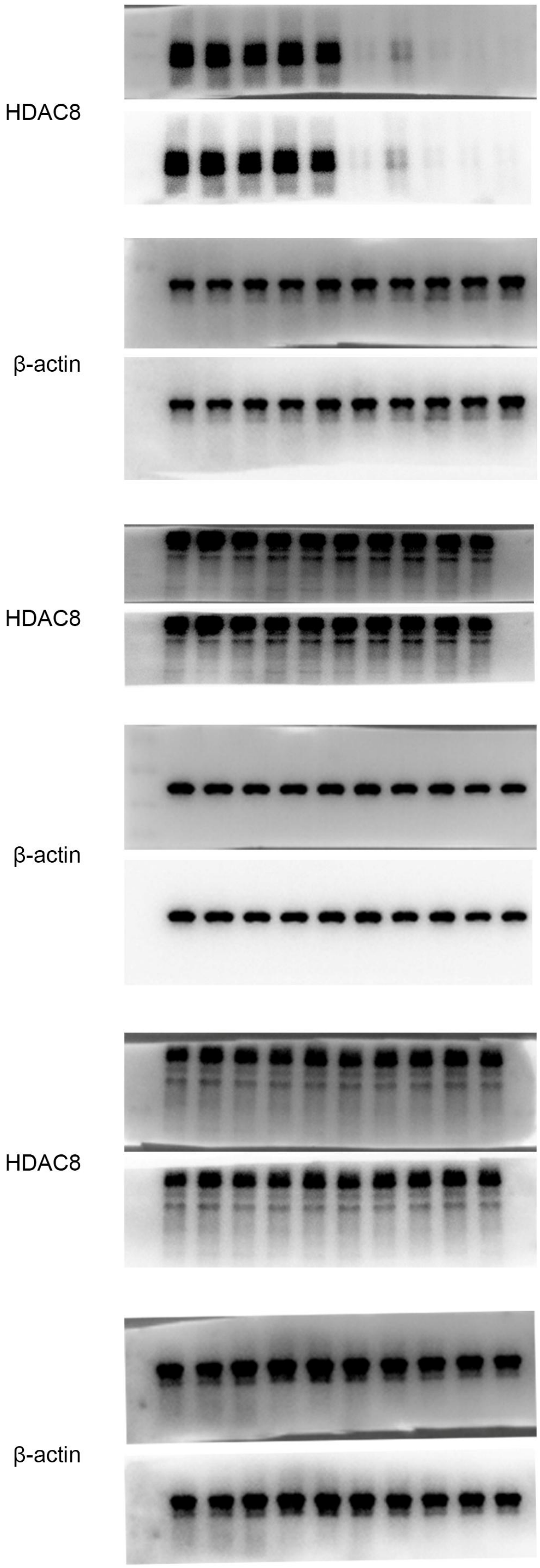

**Supplementary figure 12**

**c**

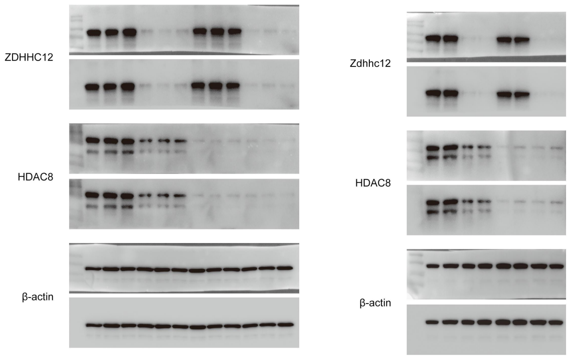


**e**

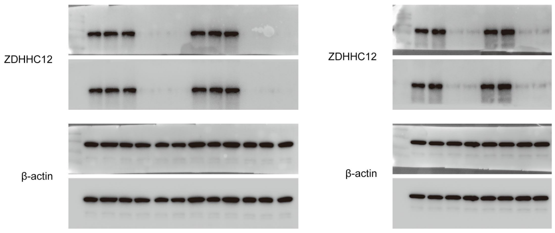

Supplement: Supplementary file 2 — Supporting Information [file ADVS-12-e05702-s001.pdf]
